# A novel strategy to block mitotic progression for targeted therapy

**DOI:** 10.1016/j.ebiom.2019.10.013

**Published:** 2019-10-25

**Authors:** Junlong (Jack) Chi, Hongchun Li, Zhuan Zhou, Javier Izquierdo-Ferrer, Yifan Xue, Cindy M. Wavelet, Gary E. Schiltz, Bin Zhang, Massimo Cristofanilli, Xinghua Lu, Ivet Bahar, Yong Wan

**Affiliations:** a Department of Obstetrics and Gynecology, Northwestern University Feinberg School of Medicine, USA; b Department of Pharmacology, Northwestern University Feinberg School of Medicine, USA; c Robert H. Lurie Comprehensive Cancer Center, Northwestern University Feinberg School of Medicine, USA; d Chemical of Life Processes Institute, Northwestern University, USA; e Department of Biomedical Engineering, Northwestern University, USA; f Department of Computational and Systems Biology, University of Pittsburgh School of Medicine, USA; g Center for Molecular Innovation and Drug Discovery, Northwestern University, USA; h Department of Biomedical Informatics, University of Pittsburgh School of Medicine, USA; i Department of Medicine-Hematology and Oncology, Robert H. Lurie Comprehensive Cancer Center, Feinberg School of Medicine, Northwestern University, USA

**Keywords:** Cdc20 PROTAC, Cdc20-APC/C, Mitotic regulation, Ubiquitination and anticancer therapy

## Abstract

**Background:**

Blockade of mitotic progression is an ideal approach to induce mitotic catastrophe that suppresses cancer cell expansion. Cdc20 is a critical mitotic factor governing anaphase initiation and the exit from mitosis through recruiting substrates to APC/C for degradation. Results from recent TCGA (The Cancer Genome Atlas) and pathological studies have demonstrated a pivotal oncogenic role for Cdc20-APC/C in tumor progression as well as drug resistance. Thus, deprivation of the mitotic role for Cdc20-APC/C by either inhibition of Cdc20-APC/C activity or elimination of Cdc20 protein via induced protein degradation emerges as an effective therapeutic strategy to control cancer.

**Methods:**

We designed a proteolysis targeting chimera, called CP5V, which comprises a Cdc20 ligand and VHL binding moiety bridged by a PEG5 linker that induces Cdc20 degradation. We characterized the effect of CP5V in destroying Cdc20, arresting mitosis, and inhibiting tumor progression by measuring protein degradation, 3D structure dynamics, cell cycle control, tumor cell killing and tumor inhibition using human breast cancer xenograft mouse model.

**Findings:**

Results from our study demonstrate that CP5V can specifically degrade Cdc20 by linking Cdc20 to the VHL/VBC complex for ubiquitination followed by proteasomal degradation. Induced degradation of Cdc20 by CP5V leads to significant inhibition of breast cancer cell proliferation and resensitization of Taxol-resistant cell lines. Results based on a human breast cancer xenograft mouse model show a significant role for CP5V in suppressing breast tumor progression.

**Interpretation:**

CP5V-mediated degradation of Cdc20 could be an effective therapeutic strategy for anti-mitotic therapy.

## 1 Introduction

The cell-division cycle protein 20 (Cdc20) is a substrate receptor of anaphase-promoting complex (APC) or cyclosome, (APC/C), which orchestrates the initiation of anaphase and the exit from mitosis through time-dependent degradation of securin and cyclin B [1], [2], [3]. In addition to its mitotic function, recent studies have linked Cdc20-APC/C to a variety of cellular processes beyond cell cycle, including apoptosis, neurogenesis, stem cell expansion and epigenetic regulation [2], [3], [4]. Malfunction of Cdc20-APC/C often result in chromosomal instability that could further lead to human diseases or predispose normal cells to become malignant [2], [3]. Results from TCGA (The Cancer Genome Atlas) and pathological analysis have revealed a strong connection between aberrant upregulation of Cdc20 and various types of cancers [5], [6], [7], [8]. For instance, severe accumulation of Cdc20 was detected in breast cancer cells and colon cancer cells compared to normal epithelial cells and adjacent normal tissues in cancer patient specimens. Immunohistochemistry analysis further described elevated Cdc20 and securin as the hallmark of a triple-negative breast cancer (TNBC) with a short survival time window for breast cancer patients [7]. Moreover, Cdc20 upregulation was associated with aggressive tumor progression and poor prognosis in gastric cancer [5] and primary non-small cell lung cancer [8]. Surprisingly, an increased expression of Cdc20 was detected in metastatic liver tissue in patients with colorectal cancer [6]. In addition, overexpression of Cdc20 was measured with a high grade in bladder, cervical, colon, endometrial, gastric, liver, ovarian, prostate, and renal carcinomas [9]. Thus, Cdc20 is thought to be a potential biomarker and an ideal target for cancer therapy.

**Resarch in context**

*Evidence before this study*

Blockade of mitotic progression is an efficient and classic approach to induce mitotic catastrophe that suppresses cancer cell expansion. Cdc20 plays an essential role in anaphase initiation and the exit from mitosis by recruiting substrates to E3 ligase for degradation. Results from recent TCGA and pathological studies have demonstrated a pivotal oncogenic role for Cdc20-APC/C in tumor progression as well as drug resistance. Although anti-Cdc20 small molecule inhibitors have been developed, the limited potency restricts their further *in vivo* application.

*Added value of this study*

Results from our TCGA and pathological analyses demonstrated the abnormal elevation of Cdc20 in breast cancer tissue, especially in triple negative breast cancer. In contrast to the traditional methodology for developing small molecule inhibitors, we employed a protein degradation-targeting therapeutic strategy and developed a potent Cdc20 degrader to destroy Cdc20 in triple negative breast cancer cells, which provides a potential valuable approach to treat breast cancer.

*Implications of all the available evidence*

Our developed CP5V compound specifically and potently degrades Cdc20 implying a new strategy for anti-mitotic therapy.

Alt-text: Unlabelled box

Blockade of mitotic progression is an effective approach to induce mitotic catastrophe to kill cancer cells [10], [11], [12]. The inhibition of several steps during mitosis, such as mitotic checkpoint, microtubule polymerization, chromatid segregation, cytokinesis and the exit from mitosis, have been considered to develop anti-mitotic drugs [11]. A series of mitotic drugs and mitotic inhibitors have been developed, including paclitaxel and inhibitors of Aurora kinase A/B, Cdk1, CENP-E, Eg5 and Polo-like kinase [11]. Because of the critical role of Cdc20-APC/C in controlling chromatid separation and the exit from mitosis, Cdc20-APC/C has been thought to be a pivotal target for drug development. The endeavor of screening for small molecule inhibitors to suppress the activity of Cdc20-APC/C has led to the development of several APC inhibitors such as TAME (tosyl-l-arginine methyl ester), proTAME (cell-permeable prodrug of TAME), apcin, and apcin-A [13], [14], [15]. While these APC inhibitors have shed light on how to efficiently block APC activation in cell-free assays, some challenges, such as efficacy in inhibiting mitosis and suppressing cancer cell growth *in vivo,* limit their current potential for clinical translation. Hence, the development of more efficient Cdc20-APC/C inhibitors or the development of new strategies to target Cdc20 are urgently needed to enhance cancer therapeutics via mitotic catastrophe.

Proteolysis Targeting Chimeras (PROTACs) are heterobifunctional molecules that can work as a bridge to form a ternary complex between a target protein and an E3 ubiquitin ligase, thereby inducing target ubiquitination and subsequent proteasome-dependent degradation [16]. The notable successes of designed PROTAC molecules in efficiently targeting estrogen receptor and androgen receptor for degradation have demonstrated their great potential to manage difficult targets such as c-Myc, K-Ras or Cdc20 [17]. There have been more than twenty kinds of protein successfully targeted for degradation [18], [19], [20], [21], [22], [23], [24]. Given the importance of Cdc20-APC/C in carcinogenesis and the current challenges for developing potent inhibitors, we have utilized the PROTAC platform to design a new targeting approach by promoting the degradation of Cdc20 in cancer cells. Based on the molecular properties of specific binding interactions between Cdc20 and apcin-A, we have designed a Cdc20 ligand in combination with various binding moieties, such as VHL (von Hippel-Lindau) and CRBN (Cereblon) [14], [25], [26], [27]. To achieve high targeting efficiency, we have further designed a series of chemical linkers that bridge the Cdc20 ligand and E3 ligase binding moiety [28]. We have identified CP5V (apcin-A-PEG5-VHL Ligand 1) as an efficient Cdc20 PROTAC. In both cultured-cell and preclinical breast cancer models, we demonstrated that the degradation of Cdc20 by CP5V induces mitotic inhibition that, in turn, suppresses cancer cell proliferation. In addition, we observed that CP5V could overcome the mitotic slippage, which is the leading cause of drug resistance to taxane chemotherapy in breast cancer. We anticipate that CP5V-mediated Cdc20 degradation could potentially provide an alternative solution to target the oncogene Cdc20 and address the Cdc20-dependent resistance to taxanes in breast cancers.

## 2 Materials and methods

### 2.1 Cell lines, antibodies, and reagents

MCF-7, U2OS, and MDA-MB-231 cells were purchased from ATCC. The MDA-MB-435 cells were kindly shared by Dr. Dinghua Yu at the University of Texas, TX. The Flag-Cdc20 MDA-MB-231 stable cells were prepared by our lab member Dr. Cindy Mandy Wavelet. The 22Rv1, and LNCaP cells were kindly shared by Dr. Jingdan Yu at Northwestern University. MCF-7, MDA-MB-231 cells were cultured in Dulbecco's Modified Eagle Medium (DMEM) supplemented with 10% fetal bovine serum (FBS) and 1% penicillin-streptomycin. Flag-Cdc20 MDA-MB-231 cells were cultured in complete DMEM introduced before with 1 μg/ ml tamoxifen. Following ATCC instruction, the U2OS cells were cultured in McCoy's 5A Medium with 10% FBS and 1% antibiotics. 22Rv1 and LNCaP cells were cultured in RPMI-1640 Medium. The Cdc20, cyclin B and actin antibodies were purchased from Sigma. The actin antibodies were diluted 1:5000. The Cdc20, cyclin B and ubiquitin antibodies were diluted 1:1000. The agarose beads were purchased from Cell Signaling Technology.

### 2.2 Western blotting

Cells were lysed in RIPA buffer (50 mM Tris (pH 7.5), 150 mM NaCl, 1% NP-40, 0.5% sodium deoxycholate, 0.1% SDS) supplemented with protease (11,697,498,001, Roche) and phosphatase inhibitors (10 mM sodium fluoride, 10 mM β-glycerophosphate, 2 mM sodium orthovanadate and 10 mM sodium pyrophosphate) for 10 min and heated at 95℃ for 5 min. The lysates were analyzed by SDS/PAGE. Western blotting was performed by transferring samples onto a nitrocellulose membrane, incubating in 5% milk in TBST (Tris-buffered Saline with Tween 20) at room temperature for 1 h and probing with indicated antibody overnight at 4 °C. Membranes were visualized using the ECL prime western blotting detection reagent (GE Healthcare, RPN2232).

### 2.3 Flag-Cdc20 pull-down assay

The Flag-Cdc20 MDA-MB-231 stable cells were treated with 5 μM of DMSO, apcin or CP5V with 10 μM of proteasome inhibitor MG-132 for 8 h at 37 °C. Cells were then lysed on ice in 50 mM Tris-HCl (pH 7.5), 150 mM NaCl, 1 mM EDTA, 1% NP-40, 10% glycerol, supplemented with protease (11,697,498,001, Roche), phosphatase, (10 mM sodium fluoride, 2 mM sodium orthovanadate, and 10 mM β-glycerophosphate) and deubiquitinase inhibitors (10 mM N-ethylmaleimide, 20 μM PR-619) for 10 min. The cells were collected by scratch with a scraper and then centrifuged at 15,000 RPM for 10 min. Equal amounts of supernatants were then incubated with 50 μL pre-washed Anti-Flag agarose beads at 4 °C for 2 h. Beads were then collected by centrifugation (5000 RPM for 1 min), washed and re-suspended in 2 × SDS buffer. Beads were then boiled for 5 min and analyzed by SDS/PAGE. Western blotting was performed according to standard protocols.

### 2.4 Cell proliferation assays

Cells were seeded in 96-well plates at the concentration of 3000 cells per well in 180 μl of media and incubated at 37 °C for 3 days. MDA-MB-231, MDA-MB-435 and MDA-MB-435 eb cells were seeded in phenol red free DMEM + 10% charcoal-stripped FBS (Omega). After overnight incubation, cells were treated with 20 μl of 10 × concentrated compound to yield indicated concentrations for each experiment. Treated cells were incubated at 37 °C for 3 days after which reagents were added to plates. After 3 days, the media with treatments were dumped and 100 μl of phenol red free DMEM with 10% CCK8 were added in each well and incubated for 1 h The absorbance of light with wavelength of 450 nm was measured with 800TS Absorbance Reader from the Biotech. The reading was normalized with respect to DMSO-treated cells, and IC50 values were calculated by nonlinear regression analysis using GraphPad Prism 6 software.

### 2.5 RNA extraction and RT-PC/R

Total RNA was extracted from breast cancer cells using Trizol and used in first-strand cDNA synthesis with High-Capacity cDNA Reverse Transcription from Thermo Scientific for RT-PCR according to the manufacturer's instructions. Each treatment was performed independently in triplicate. The PCR primers used to amplify the Cdc20 genes are Cdc20 forward primer 5′-AAT GTG TGG CCT AGT GCT CC-3′ and Cdc20 reverse primer 5′-AGC ACA CAT TCC AGA TGC GA-3′ (INTEGRATED DNA TACHNOLOGIES, USA). qRT-PCR was performed with StpeOnePlus Real-Time PCR System from Thermo Scientific. Hierarchical Clustering and combined analysis were performed using homemade scripts.

### 2.6 Flow cytometry analysis

MDA-MB-231 and MDA-MB-435 cells were plated in a 6-cm dish and double-synchronized for 18 and then 18 h with a 9-hour release with fresh medium between them. The cells were released with free medium for 9 h and treated with DMSO, apcin, proTame, Control CP5V and CP5V at indicated concentrations for 24 h. After treatment, the cells were collected and stained with propidium iodide (PI) staining buffer and analyzed by flow cytometry.

### 2.7 SPR analysis

The binding affinity of CP5V was measured using a four channel Reichert surface plasmon resonance (SPR) instrument (Reichert Technologies).  The purified His-tagged Cdc20 protein at 0.3 μM in PBS was immobilized via standard amino coupling on a 500,000 Da carboxymethyl dextran hydrogel surface sensor chip (Reichert Technologies, Depew, NY). The CP5V dissolved in PBS with 1% DMA at indicated concentrations was injected over the chip at a flow rate of 25 µl/min. Binding was detected as a change in the refractive index at the surface of the chip, as measured by response units (microRIUs). A reference flow channel was used to record the background response, and background was subtracted from each sample injection. Equilibrium dissociation constant (*K*_D_) values were calculated as ratios of association rate (*k_a_*) to dissociation rate (*k_d_*) determined from kinetic experiments. Data was fitted using TraceDrawer data analysis software available through Reichert Technologies.

### 2.8 Animal experiments

For 4T1 spontaneous mice model, 5 × 10^5^ 4T1 cells in PBS were injected into the Balb/C mice mammary fat pad. When the tumors were palpable, their (volume) growth was measured for 3 weeks and calculated using the ellipsoid equation with width (W) and length (L): W^2^ × *L* × 0.52. 10 days after 4T1 injection, 100 mg/kg CP5V (dissolved in 30% N, N-dimethylacetamide + saline) or placebo (30% N, N-dimethylacetamide + saline) were intraperitoneally administrated twice a week. The mice were sacrificed at the end of the treatment and the xenograft tumors were collected and fixed with formalin, paraffin embedded, and sectioned. Xenograft tumor and liver tissues were stained with hematoxylin and eosin, Cdc20, cyclin B1, Ki67, Activate and caspase-3. The animal study was approved by the Institutional Animal Care and Use Committee (IACUC) from Northwestern University.

### 2.9 Chemical synthesis

Apcin was purchased from Sigma-Aldrich. Apcin-A and other anti-Cdc20 PROTACs were synthesized in-house. The detailed procedure for CP5V synthesis can be found in the Supplementary Method. All compounds were prepared in DMSO stock solution at a concentration of 10 mM and stored at −20°C before use. The final DMSO concentration was less than 0.1% in the working buffer.

### 2.10 Statistical analysis

The significance of the differences in the assays was analyzed by Student's *t*-test or one or two-way ANOVA, followed by Tukey's multiple comparisons test. A *p*-value lower than 0.05 was considered to be significantly different.

### 2.11 Structural modeling and MD simulations

The structures of CP5V (apcin-A-PEG5-VHL1) and VHL-CP5V-Cdc20 complex were generated using as templates the Protein Data Bank (PDB) structures with ids 5T35 [29] and 4N14 [14] for the VHL1 and apcin-A fragments, respectively. To relieve the clashes between VHL and Cdc20, the modeled complex structure was simulated with NAMD [30] using conventional equilibration (of 4 ns) and molecular dynamics simulation protocols, and CHARMM36 [31] force field. Six runs were generated by adopting different rotational isomeric states of a C—C bond in the PEG5 linker, each varying by 60°. The VHL and Cdc20 were originally positioned in different orientations with no atom-atom contacts.

## 3 Results

### 3.1 Targeting Cdc20-APCC could be an effective approach for anti-mitotic therapy

Cdc20-APC/C is a master regulator governing mitotic progression through orchestrating chromatid separation and the exit from mitosis. Upon associating with APC/C, Cdc20 acts as a substrate receptor, which bridges securin and cyclin B to APC/C for destruction during mitosis [3]. Dysregulated Cdc20/APC/C have been reported in a variety of cancers, including gastric, lung, cervical, endometrial, liver, ovarian and breast cancers [5], [6], [7], [8]. The relatively high expression of Cdc20 has been regarded as a clinicopathological parameter in many types of human cancers as well [3]. Therefore, Cdc20 is perceived as a promising therapeutic target for cancer therapy.

**Fig. 1:**
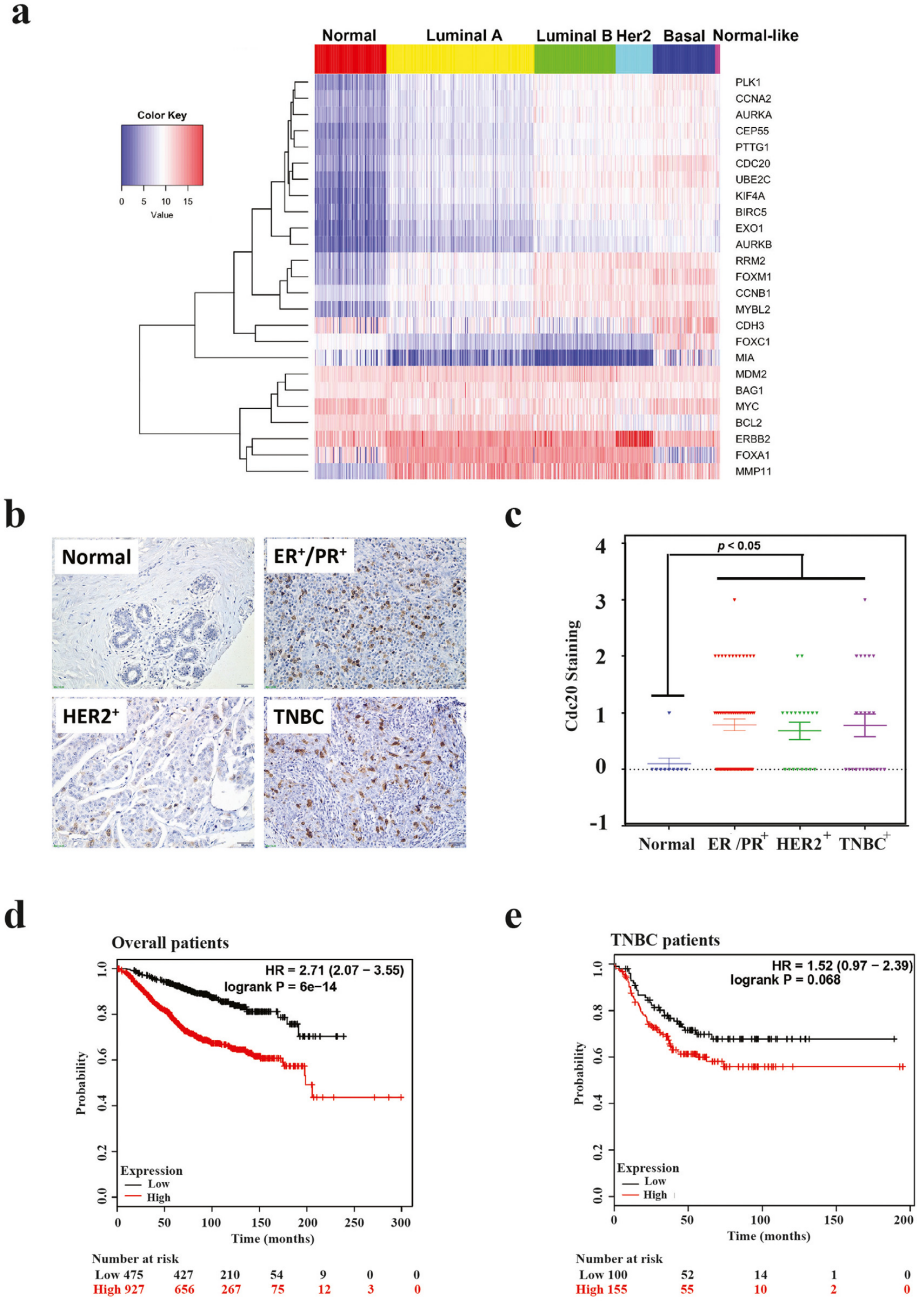
Aberrant Cdc20 expression correlates with poor prognosis of breast cancer. (**a**) Heatmap of the expression profile of Cdc20, Cdc20-APC/C pathway related genes, and other classic breast cancer genes. The data from TCGA was analyzed by hierarchical clustering, and red and blue colors represent higher and lower expression levels, respectively. The expression level of Cdc20 and its related genes and other critical breast cancer factors were compared between normal samples and the five PAM50 BRCA subtypes. (**b**) Representative Cdc20 staining in human breast cancer and adjacent normal tissues. Tissues were stained for immunohistochemical analysis as described in Materials and Methods. Normal, adjacent normal breast tissues; ER^+^/PR^+^, estrogen receptor or progestin receptor positive breast cancer tissues; HER2^+^, HER2 positive breast cancer tissues; TNBC, triple-negative breast cancer tissues. (**c**) Statistical analysis of the average score of Cdc20 staining among ER^+^/PR^+^, HER2^+^ and TNBC breast cancer tissues and corresponding non-tumor tissues, *p*-value < 0.05 (one-way ANOVA). (**d-e**) Survival graphs of breast cancer patients in relation to Cdc20 expression. Using publicly database (KM plotter; www.kmplot.com), relapse-free survival curves in overall breast cancer patients, patients with chemotherapy and patients with TNBC were analyzed for two subsets of patients, with high (red) and low (black) Cdc20 expression. HR (hazard ratio) and Log-rank *p*-values were calculated in KMplot database (For interpretation of the references to color in this figure legend, the reader is referred to the web version of this article.).

To study the clinical impact of Cdc20-APC/C in various subtypes of breast cancer, we compared the expression levels of Cdc20, Cdc20-APC/C pathway-related genes, and other classic breast cancer genes between normal tissue and various molecular subtypes of breast cancers, using expression data from TCGA database. We also applied hierarchical clustering on the genes to see if functionally related genes were clustered together. The heatmap in Fig. 1a indicates that the expression of Cdc20 in breast cancer tissues is significantly higher compared to that in normal tissue (*t*-test *p*-value < 2.2e-16). The comparison among five subtypes of breast cancer (Fig. 1a) further reveals the subtype-dependent expression pattern of Cdc20. The basal-like breast cancer, the most aggressive and metastatic subtype, exhibits the highest average expression level of Cdc20 (10.75), while the luminal A subtype has the lowest average expression level (8.20). As further confirmation of the importance of Cdc20 in breast cancer, we performed immunohistochemical analyses of Cdc20 with human breast specimens and observed severe Cdc20 protein accumulation in luminal A/B, Her2 and TNBC tissues in comparison with the adjacent normal tissues (Fig. 1b and c).

While the prognosis and survival outcomes show significant variation among different clinical subtypes, we then trained a Cox Regression model to investigate the correlation between Cdc20 expression and clinical outcomes, using the expression data and survival data from a study by the Molecular Taxonomy of Breast Cancer International Consortium (METABRIC). We used the METABRIC dataset instead of the TCGA dataset as it provides relatively more complete survival data of close to 2000 breast cancer patients. When we included Cdc20 as the only covariate, the coefficient of the expression level of Cdc20 contributing to the clinical outcome in the resulting Cox model turned out to be 0.32354. This significant positive coefficient (Wald test *p*-value < 0.001) demonstrates the positive correlation between Cdc20 expression levels and poor prognosis. Moreover, the results from Kaplan-Meier analysis indicated that patients with high expression of Cdc20 showed significantly shorter metastasis-free survival time in comparison with patients with low Cdc20 expression (Fig. 1d and e). These results demonstrate a strong connection between aberrant Cdc20 expression and poor prognosis of breast cancer and further suggest that targeting Cdc20 could be a good strategy for anti-mitotic therapy.

### 3.2 PROTAC, a novel strategy to eliminate oncogenic proteins, sheds light on the effect of targeting Cdc20-APC/C

Blocking mitotic exit has been regarded as a promising strategy to induce tumor cell death [10], [11], [12]. Owing to the pivotal role of Cdc20 in both mitotic exit and carcinogenesis, the development of small molecule inhibitors targeting Cdc20 to suppress Cdc20-APC/C activity has attracted substantial attention in the field of cancer therapy. The most common inhibition strategy is to either block the substrate binding site of Cdc20 or disrupt the interaction between Cdc20 and APC/C, or both [10]. Dr. Randall W. King discovered the Cdc20 small molecule inhibitors apcin (APC inhibitor) and apcin-A, which can competitively occupy the D-box binding pocket of Cdc20 to inhibit the ubiquitination of Cdc20 substrates, as well as small molecule inhibitors that antagonize the APC/C-Cdc20 interaction, TAME and proTAME [2], [3], [13], [15]. Apcin and proTAME efficiently inhibit the degradation of APC/C substrates and mitotic exit by simultaneously disrupting the protein-protein interactions involved in the APC/C-Cdc20-substrate ternary complex [14].

**Fig. 2:**
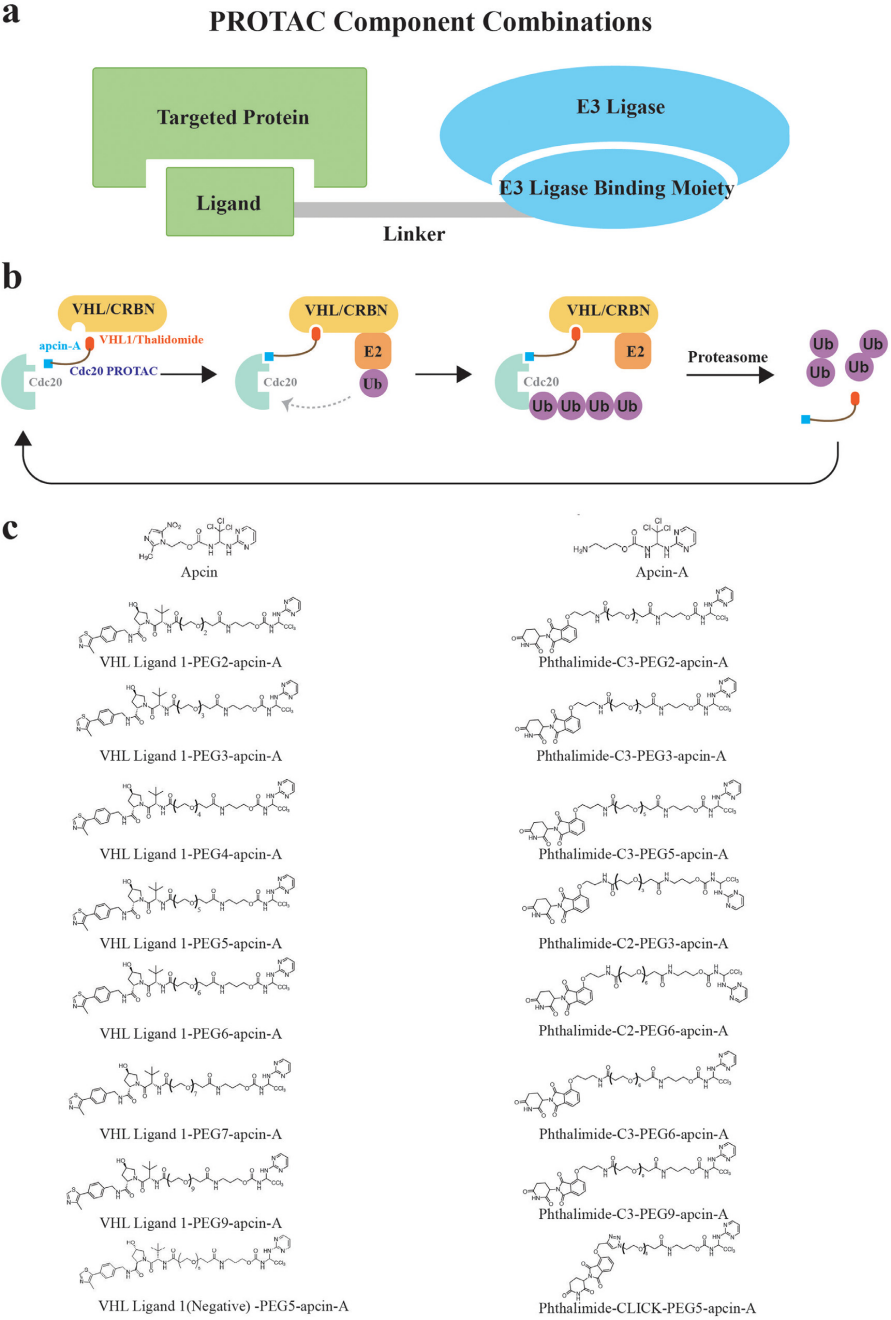
Design and selection of Cdc20 PROTACs (**a**) The mechanism of PROTAC technology is to recruit an endogenous ubiquitin protein ligase in order to induce the ubiquitination of targeted proteins for its degradation. Bifunctional PROTAC molecules bind to the targeting protein with one end while the other end binds an E3 ligase to form a ternary complex. The recruited E3 ligase then mediates the transfer of ubiquitin from an E2 enzyme to the targeting protein. The ternary complex dissociates, and the ubiquitinated targeting protein is removed by the proteasome. (**b**) The Flowchart of Cdc20 PROTACs mechanism. Apcin-A is utilized as Cdc20 targeting ligand, and VHL and CRBN binding moieties VHL1 and thalidomide are respectively used to recruit the VHL/VBC complex and Celebron E3 ligase in the Cdc20 PROTACs. A series of polyethylene glycol (PEG) molecules were used to link apcin-A and VHL1/thalidomide. (**c**) The structure of apcin, apcin-A and series of Cdc20 PROTACs designed in the present study. .

PROTACs have recently proven to have extraordinary potential as cancer therapeutics through exceptional success at degrading estrogen and androgen receptors [17], [20], [22]. Compared to small molecule inhibitors, a PROTAC shows several advantages, including higher potency and selectivity, irreversible degradation, and it is less likely influenced by critical mutations [32], [33]. Thus, we decided to develop a novel chimera molecule, utilizing the PROTAC platform, to circumvent the challenges met by current Cdc20 small molecule inhibitors. Based on the feature that apcin-A can efficiently bind to Cdc20 and is easier to modify, we used apcin-A rather than apcin as the warhead to target Cdc20 [14]. Regarding the E3 ligase recruited for Cdc20 ubiquitination followed by proteolysis, we considered VHL/VBC (VHL-Elongin BC) and CRBN (Celebron), both of which have reliable binding moieties (VHL ligand 1 or VHL1, and thalidomide, respectively) that have been applied in the design of several PROTAC molecules16,17,23. To search for an optimal chemical linker maximizing the formation of a stable Cdc20-PROTAC-VHL/VBC ternary complex, we have designed and tested a series of PEG-based linkers with different lengths, such as PEG2, PEG3, PEG4, PEG5, PEG6, PEG7, and PEG9. Fig. 2c shows the series of 15 PROTACs we designed with different combinations of E3 ligase ligands and linkers, and one negative control *(bottom, left)*, where VHL ligand 1 was replaced by its diastereomer resulting in deactivation of its interaction with VHL [34].

### 3.3 CP5V profoundly and specifically induces Cdc20 degradation by ubiquitin-proteasome system (UPS)

To characterize the effect of Cdc20 PROTACs, we have systematically measured the dose-dependent Cdc20 degradation stimulated by each of the 15 designed Cdc20 PROTACs in various breast cancer cells, including MCF7 and MDA-MB-231 cells. Our evaluation revealed that CP5V (apcin-A-PEG5-VHL Ligand 1) was the most potent Cdc20 PROTAC in degrading Cdc20 and suppressing cancer cell growth. Using CP5V, we have carefully conducted validation experiments and investigated the dose-dependent effect and time course for Cdc20 degradation as well as Cdc20 ubiquitination in various breast cancer cell lines.

**Fig. 3:**
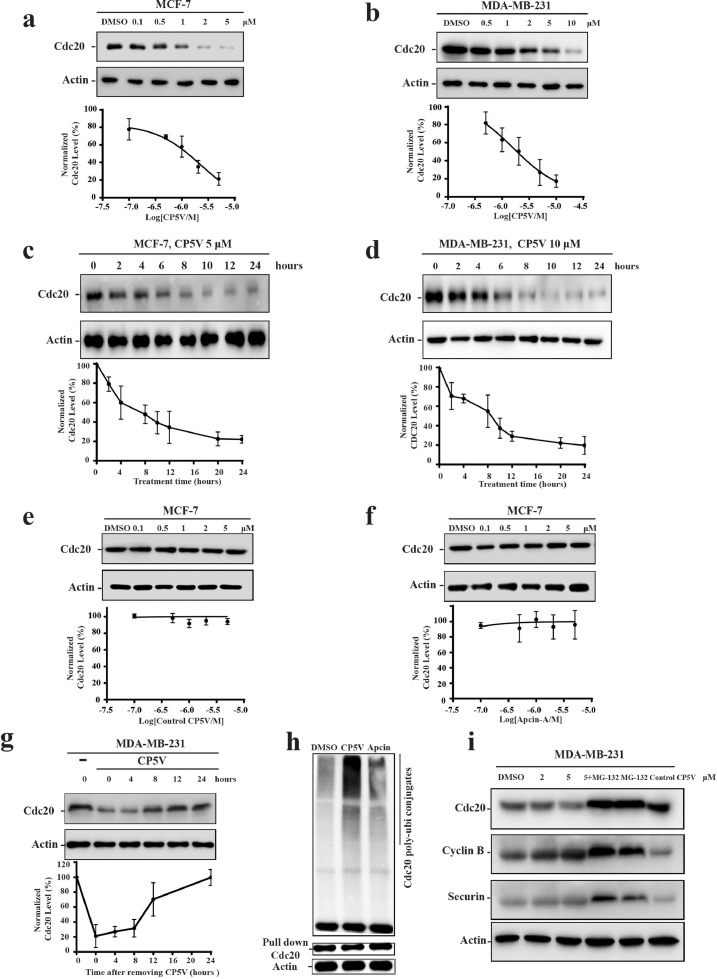
CP5V, a Cdc20 PROTAC, specifically induces Cdc20 degradation with VHL and ubiquitin-proteasome system. (**a, b**) Dosage dependency of CP5V action in various types of breast cancer cells. MCF-7 (**a**) and MDA-MB-231 (**b**) cells were treated with CP5V with indicated dosages. 10 h after the treatment, cells were collected for Cdc20 measurement. Cdc20 levels were then determined by Western blotting. (**c, d**) Time course for CP5V-induced Cdc20 degradation in MCF-7 and MDA-MB-231 cells. MCF-7 (**c**) and MDA-MB-231 (**d**) cells were treated with CP5V. Cdc20 degradation were measured at indicated time points (2, 4, 6, 8, 10, 12 and 24 h after the treatment). Cdc20 levels were then measured by Western blotting. (**e, f**) Repetitive does-dependent treatments with control PROTAC molecule. (**e**) and apcin-A (**f**) in MCF-7 cells. Neither control PROTAC nor apcin-A alone leads to degradation of Cdc20 irrespective of dosage. (**g**) The MDA-MB-231 cells were treated with CP5V at 2 μM for 8 h followed by a release in fresh medium. Cells were collected at different time points (0, 4, 8, 12, 24 h) for measuring Cdc20 protein levels by Western blotting. The assays for a-g were measured in triplicate (*n* = 3). Data are mean ± SEM. (**h**) CP5V targets Cdc20 for degradation through ubiquitination. MDA-MB-231 cells were treated with 2 μM CP5V for 6 h in the presence of 5 μM MG-132. Cdc20 were immunoprecipitated by antibody against Cdc20 followed by immunoblotting using anti-ubiquitin antibody. (**i**) MG-132 inhibits CP5V-mediated Cdc20 degradation. MDA-MB-231 cells were treated with CP5V or CP5V and MG-132 (5 μM) at the indicated dose for 10 h. Cdc20, cyclin B, securin, and actin levels were determined by Western blotting.

To test the effect of CP5V dosage, we treated both MCF-7 and MDA-MB-231 cells with CP5V for 10 h at indicated concentrations (Fig. 3a and b) and then harvested the cells for measuring their Cdc20 abundance. Our results presented in Fig. 3a and b demonstrate that CP5V profoundly degrades Cdc20 in both MCF7 and MDA-MB-231 cells with DC_50_ (CP5V concentration for 50% of maximum degradation) being approximately 1.6 μM. We have further measured the time course for CP5V-induced Cdc20 degradation in both cell lines. As shown in Fig. 3c and d, we observed that the half-life for Cdc20 in the presence of CP5V is approximately 4 h in both MCF-7 and MDA-MB231 cells. We did not observe apparent recovery of suppressed Cdc20 protein levels for 24 h (Fig. 3c and d).

After establishing the potency of CP5V in Cdc20 degradation, we next examined if the observed CP5V-induced Cdc20 degradation was facilitated by VHL-mediated ubiquitin binding. To confirm that the CP5V-induced degradation of Cdc20 relies on the VHL E3 ligase, we designed a negative control PROTAC by replacing the VHL ligand 1 with its diastereomer, which deactivated its interaction with VHL [34]. As Fig. 3e and f indicate, we observed no decrease in Cdc20 level in the presence of negative control PROTAC, suggesting that VHL plays an essential role in the CP5V-induced Cdc20 degradation. To test how the abundance of Cdc20 would change after removing CP5V from the medium, we treated the breast cancer cells with CP5V and then removed CP5V followed replacement with fresh medium. Fig. 3g and Supplementary Figure 1a shows that the fluctuation of Cdc20 protein level recovery in 24 h after withdrawing CP5V.

To investigate whether the CP5V-induced degradation of Cdc20 is mediated through the ubiquitin-proteasome pathway, we measured the Cdc20 ubiquitination in the absence or presence of CP5V as well as apcin-A. We treated MDA-MB-231 cells with either 2 μM CP5V or 2 μM apcin-A together with or without MG-132. As shown in Fig. 3h, we observed, while apcin-A gently increased the ubiquitylation of Cdc20, CP5V significantly enhanced Cdc20 ubiquitylation. We further observed the CP5V-induced drop off Cdc20 protein abundance was attenuated by adding up MG-132 [35], confirming the fact that CP5V-induced Cdc20 degradation is mediated by ubiquitin-proteasome pathway (Fig. 3i).

VHL complex (EloB/EloC-Cul2/5-RBX1-VHL) belongs to Cullin-RING-ligase (CRL) family target HIF-α and other substrate protein for ubiquitylation [36], [37]]. Given that activation of Cullin E3 ligase is often involved in neddylation, we ask if CP5V-induced Cdc20 degradation is sensitive to neddylation inhibitor MLN4924. To date, we treated the MDA-MB-231 cells with MLN4924, which can inactivate CRLs by inhibiting cullin neddylation [38], [39], [40]. As shown in Supplementary Figure 1b, we observed the treatment of MLN4924 inhibited the degradation of Cdc20 caused by CP5V.

To further confirm the conclusion that the CP5V-medicated degradation is depending on the ubiquitin-proteasome system, we conducted RT-PCR to measure the alteration of Cdc20 RNA levels in response to CP5V treatment. As shown in Supplementary Figure 1c, we did not observe significant change of C dc20 mRNA level, confirming that the CP5V-induced Cdc20 protein level drop off is achieved through the post-translational modifications.

The off-target effect is often a concern for drug development [41], [42]. To test the specificity for CP5V in targeting Cdc20, we repeated the dosage experiment based on MDA-MB-231 cells and measured the alteration of protein levels for Cdc20 and Cdc27, another core subunit of anaphase-promoting complex [43], [44], [45], [46]. As shown in Supplementary Figure 1e, while CP5V cause obvious decrease of Cdc20, none Cdc27 protein level change was observed, suggesting that CP5V selectively incudes Cdc20 degradation. Taken together, the above characterization demonstrated that CP5V is a potent PROTAC, which can induce the targeted degradation of Cdc20 through VHL-dependent ubiquitin proteasome pathway.

### 3.4 CP5V significantly inhibits mitotic progression and induces breast cancer cell death

**Fig. 4:**
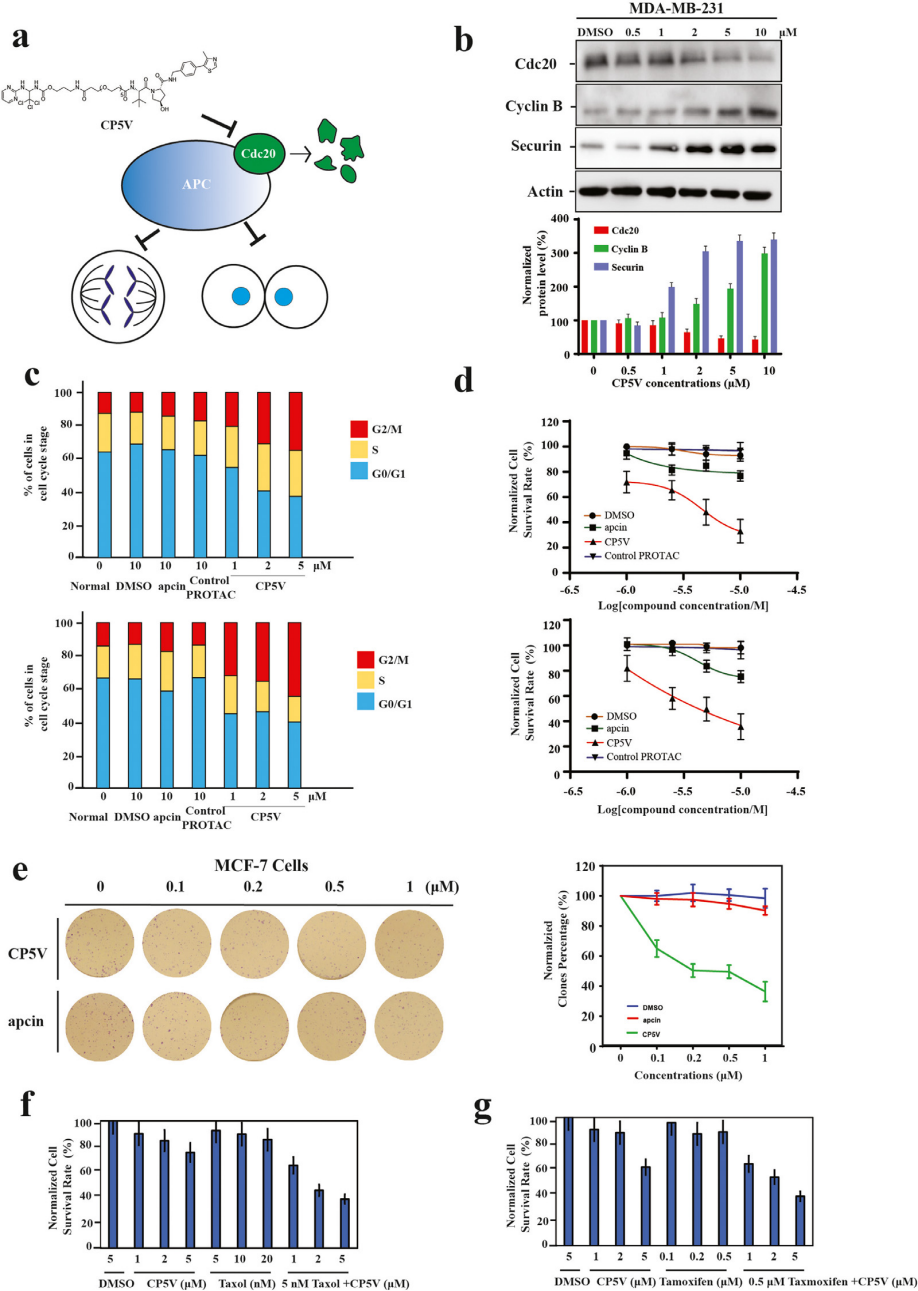
CP5V significantly inhibits mitotic progression, cancer cell growth, and further induces cancer cell death. (**a**) Schematic description of the process of targeting Cdc20 for degradation by CP5V leading to inhibition of chromatid separation and the exit from mitosis. (**b**) CP5V-mediated Cdc20 degradation results in accumulation of cyclin B. MDA-MB-231 cells were treated with CP5V at the indicated dose for 10 h. Cdc20 and cyclin B levels were then determined by Western blotting. The Western Blot is representative of 3 independent experiments (*n* = 3). Data are mean ± SEM. (**c**) CP5V induces mitotic arrest. The MDA-MB-231 (upper panel) and MDA-MB-435 cells (lower panel) were initially synchronized by double-thymidine treatment followed by a 9-hour release with fresh medium and indicated treatments for 16 h. Cell cycle profile was then measured by flow cytometry and analyzed by ModFit LT. (**d**) CP5V causes significant inhibition of cell growth in MDA-MB-231 (upper panel) and MDA-MB-435 cells (lower panel). MDA-MB-231 and MDA-MB-435 cells were plated in 96-well plates at the concentration of 3000 cells/well and treated with DMSO, apcin, Control CP5V and CP5V for 72 h and the cell survival activity was measured by CCK8 assay. The IC50 of CP5V for MDA-MB-231 cells is 2.6 μM and 1.99 μM for MDA-MB-435 cells. The test was performed in triplicate (*n* = 3). Data are mean ± SEM. (**e**) Clonogenic assay of the effect of CP5V on MCF7 cells. 200 MCF7 cells were plated in 6-well plate and treated with CP5V at gradient concentrations (0.1, 0.2, 0.5, 1 μM) for 24 h following culture for 2 weeks. To quantify the colony formation, the cells were stained with crystal violet and the numbers of colonies were counted and quantified by Image J. Left panel shows the representative clones. Right panel shows the statistical results. Data are presented as the mean ± standard error of the mean (S.E.) of three independent samples (** *p*-value < 0.01). (**f**) CP5V can restore the Taxol-induced cytotoxic response for Taxol-resistant MDA-MB-435 eb. cells and can inhibit their growth. (**g**) CP5V rescues the endocrine response for tamoxifen-resistant MCF-7 4HTR cells. The measurements were performed in triplicate (*n* = 3). Data are mean ± SEM.

APC/C-Cdc20 is a critical factor orchestrating the onset of anaphase and the exit of mitosis [4]. The knockdown of Cdc20 with siRNAs can inhibit chromatid separation and slow cyclin B1 proteolysis, which dramatically elongates the duration of cells in the mitotic phase and disturbs reconstitution of the next interphase [4], [10]. Given the fact that Cdc20-APC/C regulates mitotic progression through proteolysis of securin and cyclin B1, we measured whether CP5V-mediated degradation of Cdc20 changes the levels of securin and cyclin B1. Notably, we observed that, as Cdc20 underwent degradation, cyclin B1 and securin largely accumulated in cells (Fig. 4b). Moreover, using flow cytometry analysis, we measured the cell cycle distribution of breast cancer cells after the treatment with CP5V, and noticed that CP5V induced dramatic mitotic arrest in MDA-MD-231 and MDA-MB-435 cells, while there was no effect upon treatment with apcin alone (Fig. 4c). In the presence of 2 μM CP5V, over 35% of the MDA-MB-231 cells and 45% of MDA-MB-435 cells were arrested in the G2/M phase. The fraction of cells in the G0/G1 phase were much lower compared to that of control cells, which confirmed that the mitotic exit was restricted. Furthermore, CP5V significantly inhibited cell growth in various TNBC cell lines (Fig. 4d) with IC50 values of 2.6 μM for MDA-MB-231 cells and 2.0 μM for MDA-MB-435 cells.

In addition, we measured the efficacy of CP5V in killing breast cancer cells by using a clonogenic assay [47]. We plated MCF7 cells in the 6-well plate at the concentration of 200 cells per well and treated them with CP5V at various concentrations (0.1, 0.2, 0.5, 1 μM) for 24 h followed by culturing them for 2 weeks. To quantify the colony formation, we stained the cells with crystal violet and quantified the number of colonies by ImageJ. CP5V exhibited a high efficacy in killing breast cancer cells at 1 μM, as shown in Fig. 4e.

Relatively high potential dosage of VHL Ligand 1 offered by CP5V causes concern if it affects HIF1α, the substrate of VHL. To test the possible change of HIF1α caused by VHL ligand 1 and CP5V, we treated MDA-MB-231 cells with either VHL Ligand 1 or CP5V. As shown in Supplementary Figure 1d and e, while we observed significant HIF1α is accumulated in response to VHL ligand 1, no obvious alteration of HIF1α is observed in response to CP5V treatment.

Given Cdc20/APC plays critical role in governing chromatids segregation and overexpression of Cdc20 is linked to various types of cancers^9^, we tested the effect of CP5V in three different cancer cell lines including U2OS (osteosarcoma cell), 22Rv1 (prostate carcinoma cell) and LNCaP (prostate carcinoma cell). As shown in Supplementary Figure 3a–f, we observed CP5V showed its ability to induce Cdc20 degradation and growth inhibition in all tested cancer cell lines, suggesting the broad effect of CP5V in various cancer type cells. Collectively, our validation demonstrates that CP5V can profoundly inhibit mitotic progression and induce cancer cell growth inhibition.

### 3.5 CP5V restores Taxol-induced cytotoxic response in Taxol-resistant cells

Due to the significance of mitotic progression in cancer cell proliferation, anti-mitotic drugs like paclitaxel have been widely applied to various solid cancers, including TNBC [48], [49], [50]. Current anti-mitotic drugs including taxanes, vinca alkaloids and epothilones, principally disturb the function of microtubules, resulting in spindle assembly failure. This failure triggers mitotic catastrophe by disrupting spindle assembly checkpoints (SAC) and inducing mitotic arrest [48], [51]. However, some of the cancer cells can bypass the mitotic exit and avoid apoptosis through premature exit or rapid slippage [50], [52]. The failure to initiate apoptosis in mitotic arrest has been considered the key factor contributing to the resistance against the taxanes, since mitotic arrest without subsequent apoptosis is a common phenomenon after taxane treatments in various cancer cell lines [53]. The mitotic slippage is believed to be generated by gradual proteolysis of cyclin B [54]. Cdc20 is the co-activator of APC/C complex, which targets cyclin B for ubiquitination and degradation in normal mitosis process [4]. Thus, suppression of Cdc20 emerged as a possible strategy to overcome the drug resistance initiated by mitotic slippage. Single-cell results have confirmed that knockdown of Cdc20 by siRNA can effectively prolong the time of cancer cells to be arrested in the mitosis phase and can efficiently promote cell death during mitotic arrest [10]. This suggests the use of current PROTAC in combination with paclitaxel in order to kill apoptosis-resistant cancer cells [10].

To examine whether CP5V can overcome the taxane resistance and facilitate the taxane-induced cell death, we have examined the combinational effect of CP5V and paclitaxel against Taxol-resistant cell line MDA-MB-435 eb, whose tolerance against paclitaxel is up to 20 nM. Fig. 4f shows that about 92% of MDA-MB-435 eb cells can survive in the presence of 5 nM paclitaxel, but CP5V significantly restored their Taxol-cytotoxic response. The synergistic effect of 5 μM of CP5V and 5 nM of paclitaxel caused more than 50% of the growth inhibition of MDA-MB-435 eb cells, which made a severe difference with less than 10% of inhibition with 5 nM of paclitaxel only. Interestingly, we also observed that CP5V significantly resensitizes tamoxifen-resistant breast cancer cells to tamoxifen treatment (Fig. 4g).

### 3.6 Molecular modeling of the interaction between Cdc20 PROTAC, Cdc20 and VHL

**Fig. 5:**
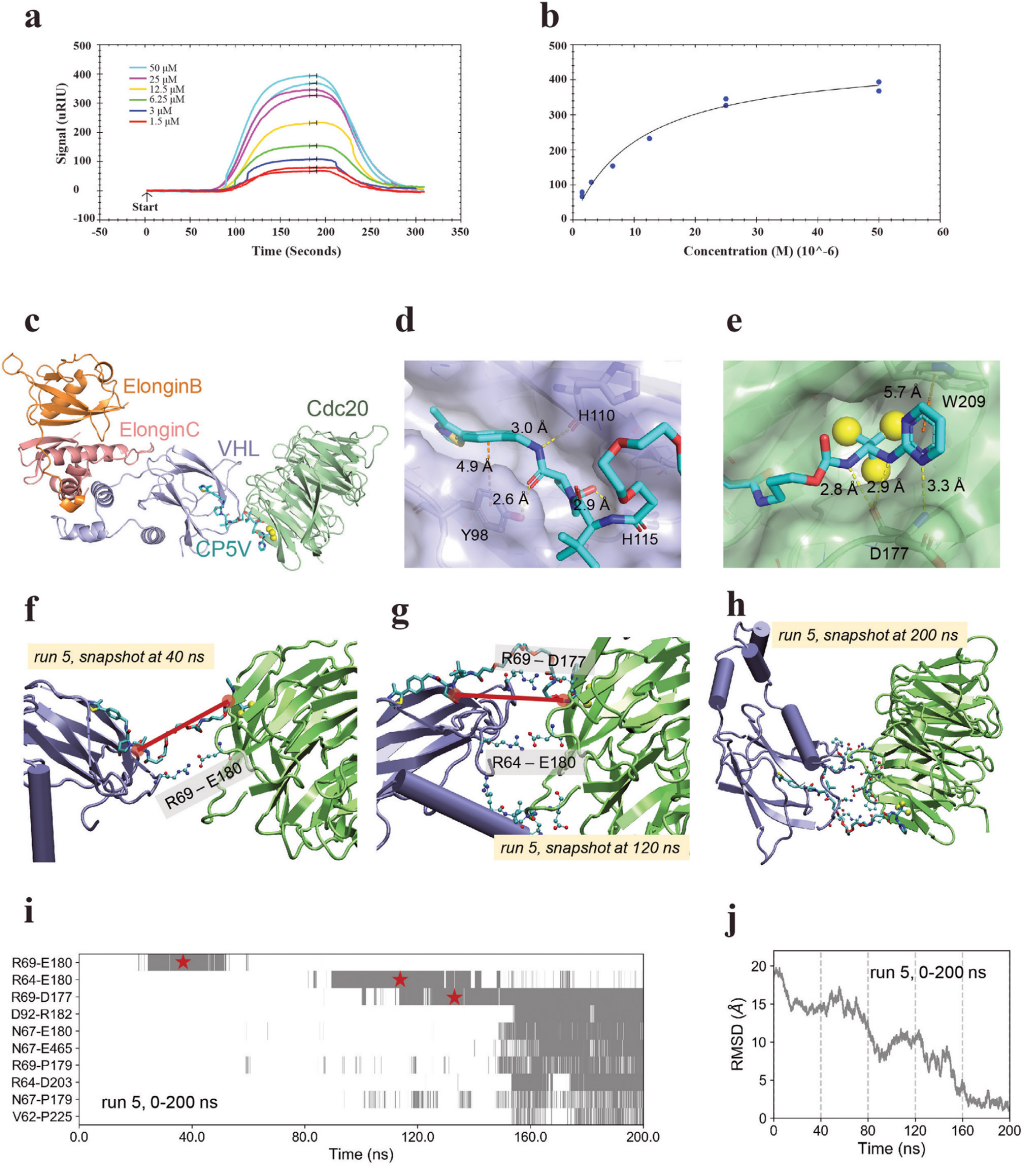
CP5V efficiently binds onto Cdc20 to help stabilize Cdc20-PROTACS-VHL-ElonginC-ElonginB complex**.** (**a**) Reference corrected SPR sensograms for CP5V binding to His-tagged Cdc20 at concentrations: 1.5 μM (*red*), 3 μM (*blue*), 6.25 μM (*green*), 12.5 μM (*yellow*), 25 μM (*magenta*) and 50 μM (*cyan*). The intervals selected for the Langmuir plot are shown as black bars. The data fits to a Langmuir binding isotherm model (solid line).  (**b**) Quantification of SPR analysis for CP5V. The *K*_D_ for CP5V is 11.2 ± 3 μM. (**c**) Modelled structure of ElonginB-ElonginC-VHL-CP5V-Cdc20 complex. The structure of Cdc20 and docking pose of apcin-A part of the CP5V are taken from the PDB structure for Cdc20 complexed with apcin (PDB id: 4N14). The apcin-A part binds to the D-box binding site of Cdc20. The structures of the complex ElonginB-ElonginC-VHL and the docking pose of the CP5V that serves as VHL ligand (VHL-1) are taken from the PDB structure for PROTAC MZ1 in complex with the bromodomain of Brd4 and VHL (PDB id: 5T35) (**d**) Close-up view of the interaction between CP5V and VHL. VHL is shown in surface representation. Closely interacting residues are labeled. (**e**) Close-up view of the interaction between CP5V and Cdc20. Hydrogen-bonds and π-π interactions between the ligand and the protein are shown by *yellow* and *orange* dashed lines, respectively. Chlorine atoms in trichloromethyl group are represented as *yellow* spheres. Transient interfaces between VHL and Cdc20 observed in snapshots at (**f**) 40 and (**g**) 120 ns during MD simulation. Interprotein salt bridges (Arg69-Glu180, Arg64-Glu180 and Arg69-Asp177), parallel to the line connecting the anchor sites (*red spheres*) on the VHL-1 and apcin-A moieties of CP5V, stabilize the transient conformations. (**h**) Equilibrated conformation of VHL-CP5V-Cdc20 ternary complex observed in snapshot at 200 ns of MD run 5. Intermolecular contact-forming residues (with any two heavy atoms closer than 4.0 Å) are shown (see also **Movie S1**). (**i**) shows the time evolution of key intermolecular contacts observed in run 5. The *gray* regions indicate the time intervals during which the contacts indicated along the ordinate have been formed and maintained. (**j)** gradual convergence from a random conformation (of the two proteins) to a stable one evidenced by the decrease in the C^α^-based root-mean-square deviations (RMSDs) with respect to the equilibrium conformation reached at 200 ns. .

Results from the above characterization demonstrate the pharmacological role for CP5V in inducing Cdc20 degradation, mitotic progression and cell death. To confirm that CP5V can actively bind onto Cdc20 to induce its degradation, we measured the binding affinity of CP5V to purified Cdc20 recombinant protein using SPR analysis. To do so, we engineered His-Cdc20 expression clone and purified His-tagged Cdc20 protein followed by SPR analysis. As shown in Fig. 5a and b, we observed that CP5V could efficiently bind onto His-tagged Cdc20 with *K*_D_ = 12.4 μM.

Toward gaining a molecular level understanding of the mechanism underlying CP5V-mediated inhibition of cancer cells growth, we conducted structure-based molecular modeling studies and molecular dynamics (MD) simulations [23], [24], [55], [56]. Our goal was to explore how CP5V stabilized the ternary complex VHL-CP5V-Cdc20. To elucidate the 3D structure of the PROTAC-induced complex, we simulated the binding of CP5V onto both VHL and Cdc20 using previously resolved substrate-binding poses: mainly, we used the structure of PROTAC MZ1 in complex with the bromodomain or Brd4 and VHL, deposited in the PDB (PDB id: 5T35) ADDIN EN.CITE to model the interaction of VHL-1 with VHL; and the crystal structure Cdc20 complexed with apcin (PDB id: 4N14) [14], to model the interaction of apcin-A with Cdc20; and we connected the two fragments by polyethylene glycol pentamer PEG5 (Fig. 5c–e). At one end, the benzyl ring of CP5V formed π-π stacking interactions with Tyr98 of VHL, and the neighboring hydroxyproline (Hyp) group formed multiple hydrogen bonds with Tyr98, His110 and His115 (Fig. 5d). At the opposite end containing the apcin-A fragment, the hydrophobic trichloromethyl group inserted into a hydrophobic pocket of Cdc20 and occupied the D-box-binding pocket of the WD40-domain [14] (Fig. 5e). Apcin-A also formed π-π interactions, this time with Trp209 of Cdc20 in addition to hydrogen bonds with backbone N and C atoms of Asp177 (Fig. 5e). The ternary complex was observed to be stably maintained in five out of six MD simulations, including one extended to 200 ns (Supplementary Figure 4). The PEG5 linker was observed to be quite flexible leading to a multitude of relative orientations of the linked proteins, Cdc20 and VHL while the apcin-A and VHL-1 fragments remained anchored to the respective proteins (Fig. 5h and Movie S1). We noted in particular the formation of interprotein salt bridges VHL–Cdc20 Arg69–Glu180, Arg64-Glu180 and Arg69-Asp177 (Fig. 5f and g and Supplementary Figure 4h) which assisted in the association of the two proteins.

It is interesting to observe that the temporarily stabilized salt bridges lied parallel to the anchor sites of the PEG5 linker in the PROTAC (Fig. 5f and g). Therefore, PROTAC not only bridged the two proteins, but restrained the transient interactions and reduced the conformational search for their effective interaction. An optimal length of the PEG linker was important to balance the enthalpic and entropic effects, and efficient sampling of conformations.

A detailed description of the frequency of inter-protein contacts and the time evolution of the contacts can be seen in Supplementary Figure 4 and Fig. 5i, and an extended simulation of 200 ns is presented in the Supplemental Movie S1, which further validated the preferred interactions and their time evolution. Examination of the pattern of gray entries in Fig. 5i clearly shows that the VHL-Cdc20 salt bridge Arg64-Glu180 forms first, followed by the persistent interprotein Arg69-Asp177 salt bridge, which drives the stabilization of several electrostatic (strong) interactions between the two proteins, including Asp92-Arg182, Asn67-Arg180, Asn67-Glu465, and Arg64-Asp203, where the first residue belongs to VHL and second, to Cdc20. Finally, such strong interactions further stabilized an equilibrated conformation of the ternary complex (Fig. 5j). Thus, the current models and simulations provide a mechanistic perspective in support of the efficacy of CP5V in enabling a close association between the two proteins. CP5V exhibits a high propensity to remain stably anchored in the Cdc20 D-box-binding pocket consistent with its potency to facilitate the ubiquitination of Cdc20 mediated by the tightly linked VHL.

### 3.7 CP5V is a potent inhibitor that suppresses breast tumor progression with no toxicity in 4T1 xenograft model

**Fig. 6:**
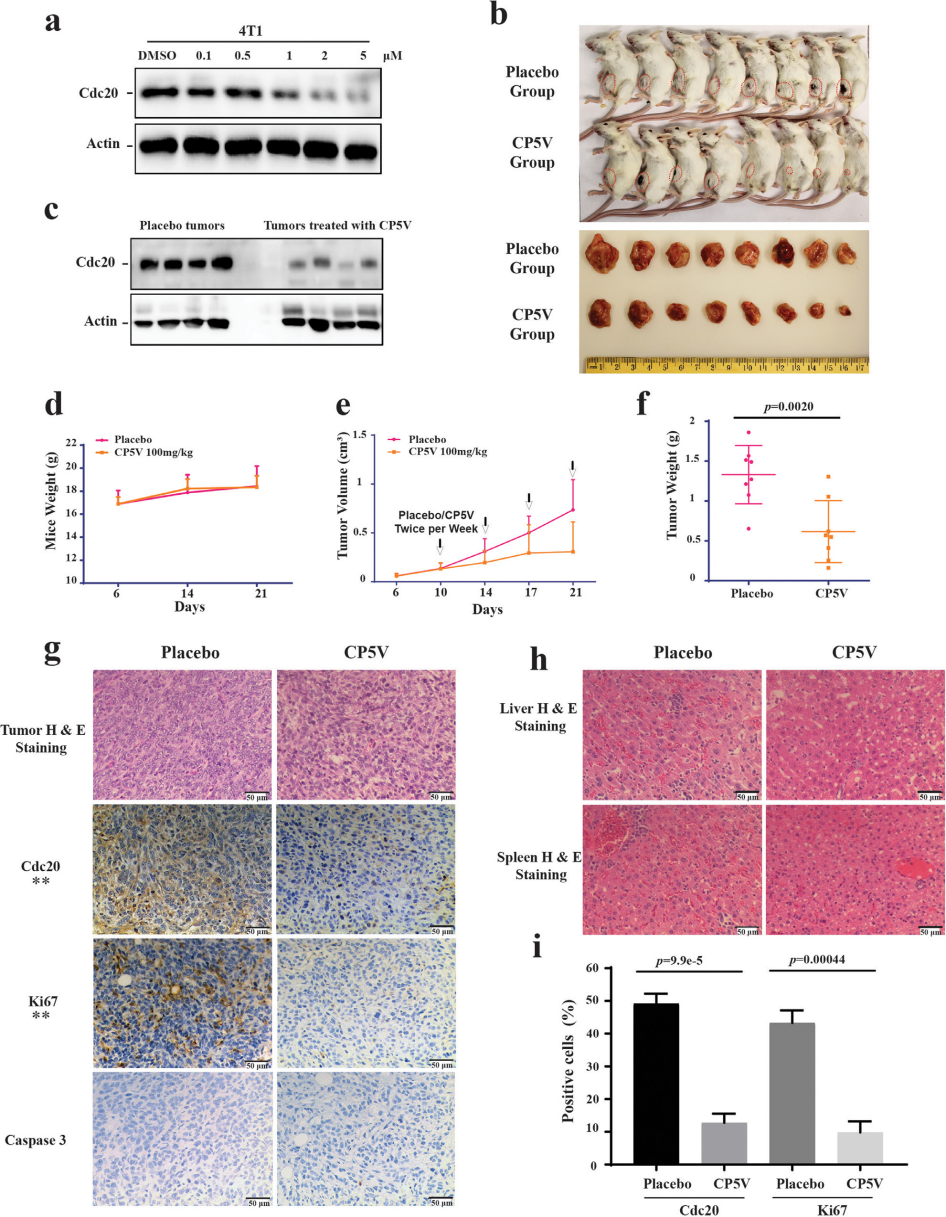
CP5V is a potent inhibitor that suppresses breast tumor progression with no toxicity in the 4T1 xenograft model. (**a**) CP5V can efficiently cause degradation of Cdc20 in 4T1 cells lines *in vitro*. (**b**-**e**) CP5V dramatically inhibits tumor growth. 4T1 cells were implanted into the mammary fat pad of BALB/c mice. Drug treatment started on the 10th day. Placebo or CP5V at the dose of 100 mg/kg was administrated twice a week for two weeks. (b) The image of 4T1 xenograft tumors harvested after 21 days. (**c**) Western blotting assays the expression of Cdc20 in 4T1 xenograft tumor. (**d**) Body weight curve of mice. (**e**) Tumor growth curve. Tumor volume was measured twice a week. The asterisk represents the significant difference (*p*-value < 0.05) between group Placebo and group CP5V. (**f**) Tumor weight curve. (**g**) Immunohistochemistry Staining of H & E, Cdc20, Ki67, and activated caspase 3 in 4T1 xenograft tumors. Scale bar, 50 μm. (**h**) H & E staining of tumor and liver of mice from both Placebo and CP5V group. (**i**) Quantification of Ki67 and activated caspase 3 positive cells in 4T1 xenograft tumors. Data are mean ± SEM.

Results based on our cultured-cell characterization have demonstrated the potency for CP5V in efficiently inhibiting mitotic progression and inducing cancer cell death. To further determine the physiological and clinical relevance of CP5V in breast cancer progression and it potential as a therapeutic agent, we have examined the impact of CP5V in mice 4T1 xenograft model. As shown in Fig. 6a, CP5V dramatically induced dose-dependent Cdc20 degradation in 4T1 cells. To build the tumor model, we injected 4T1 cells into the mammary fat pad of Balb/C mice and conducted CP5V intraperitoneal administration twice a week at the concentration of 100 mg/kg. The results demonstrated that the administration of CP5V reduced approximately 70% of the size and weight of the 4T1 xenograft tumor compared to the placebo, while no significance effect on mice body weight and liver toxicity was observed (Fig. 6b–f). Immunohistochemistry (IHC) analysis showed that CP5V dramatically decreased Cdc20 expression in the 4T1 xenograft tumor (Fig. 6g and h). We further measured the expression levels of Cdc20, Ki67 and activate caspase-3 in the isolated grafted tumors using IHC analysis, and confirmed that CP5V reduced tumor growth by decreasing the Ki67 index in the tumor (Fig. 6h and j). Altogether, our results demonstrate that the targeted degradation of Cdc20 by our newly developed Cdc20 PROTAC could be a novel strategy to treat TNBC (Fig. 6).

## 4 Discussion

**Fig. 7:**
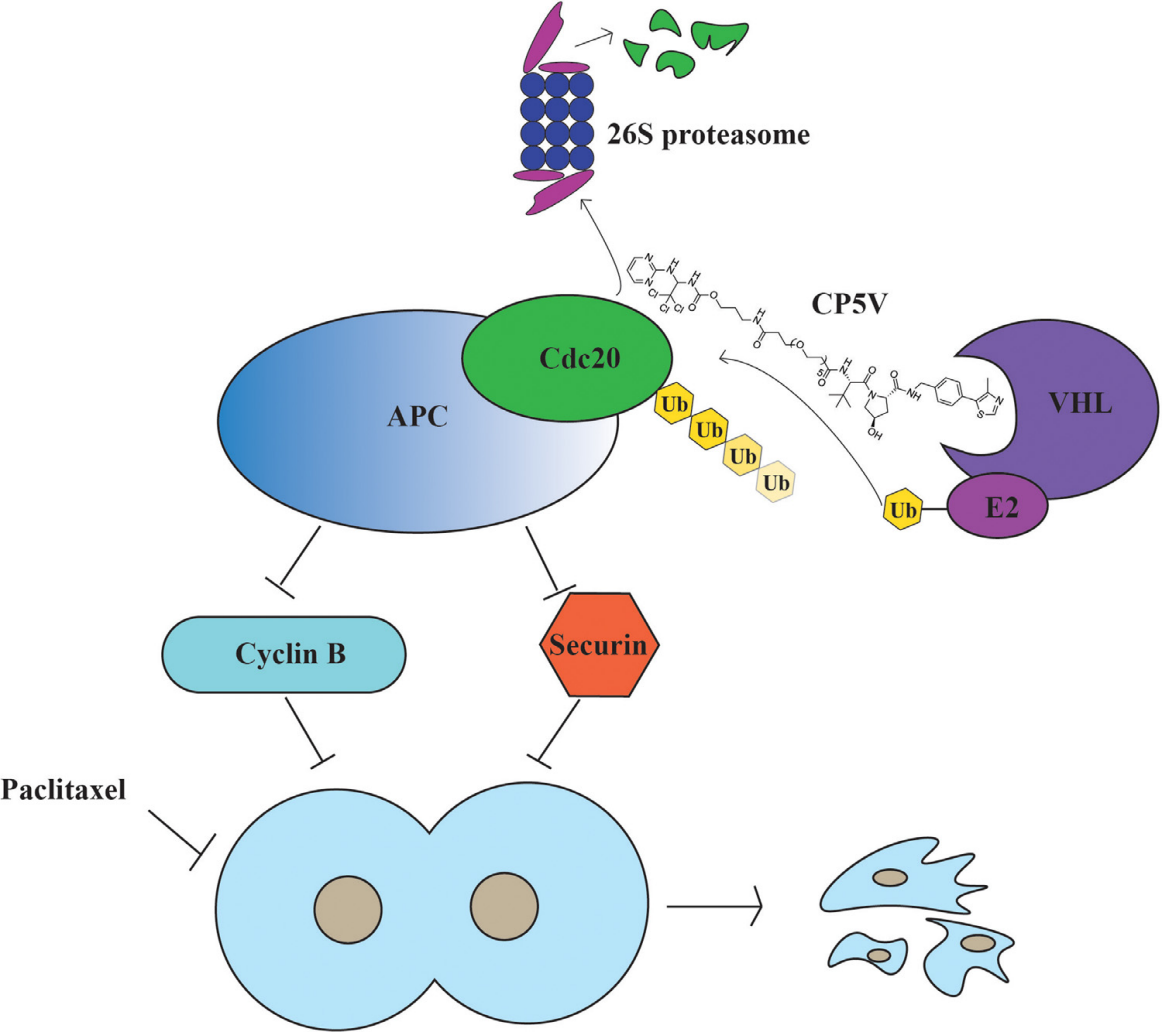
Hypothetical working model. Cdc20/APC is a master mitotic regulator governing chromatid separation and the exit from mitosis. CP5V induces specific destruction of Cdc20 by displacing Cdc20 to the close proximity of the VHL/VBC complex for ubiquitination followed by the proteasomal degradation. The CP5V-induced Cdc20 degradation leads to prolonged mitotic arrest that, in turn, results in suppression of breast cancer cell proliferation and onset of cancer cell death. Downregulation of Cdc20/APC by CP5V resensitizes drug-resistant breast cancer cells to taxane as well as tamoxifen.

Targeting mitotic catastrophe is one of the cutting-edge strategies in cancer therapy [10]. Cdc20-APC/C is a master regulator of mitosis, whose deregulation is tightly linked to various cancers [5], [6], [7], [8]. Results from pathological and TCGA studies suggest Cdc20 to be a high potential clinical target. Here we report that, based on the PROTAC platform, we have developed a potent chemical molecule named CP5V, which can induce robust Cdc20 proteolysis and inhibit cancer cell proliferation. *In vivo*, CP5V efficiently induces the formation of Cdc20-CP5V-VHL/VBC ternary complex and recruits Cdc20 to VHL/VBC E3 ligase for ubiquitination and degradation, as schematically described in Fig. 7. The destruction of Cdc20 leads to upregulation of its substrates, including securin and cyclin B [1]. The upregulation of securin and cyclin B could disturb the normal chromatid segregation and mitotic exit, which further results in mitotic arrest in breast cancer cells. Given the previously reported role of Cdc20 in tumor progression and resistance against anti-mitotic drugs, development of CP5V potentially provides a novel approach for anti-mitotic therapy.

Cdc20 has attracted considerable attention due to its potential clinical value. Development of small molecule inhibitors has been a major strategy to target Cdc20. The King laboratory has made significant contributions to the search for APC/C inhibitors with the development of several promising APC inhibitors, including apcin, apcin-A ,TAME, and proTAME [13], [14], [15]. Apcin specifically targets Cdc20, which binds to the destruction box located on substrate proteins that, in turn, prevents substrate interaction with Cdc20 and ubiquitination by Cdc20-APC/C [14]. Apcin-A is an improved apcin derivative obtained upon elimination of the nitro-imidazole moiety, which makes it more accessible for modification [14]. TAME specifically blocks APC/C activation by directly binding to APC/C and disrupts the IR-tail-dependent interactions between APC and its activator proteins Cdc20 or Cdh1 [15]. It stabilizes APC substrates and has been shown to inhibit cyclin proteolysis in mitotic Xenopus egg extract with an IC_50_ of 12 µM. Since TAME is not cell permeable, proTAME has been designed to be processed by the intracellular esterase to produce the parent compound [13]. Apcin could enhance the effect of TAME, leading to an increase in the number of cells in mitosis, a longer mitotic duration, and greater stabilization of cyclin B1, securin, and cyclin A. Combination of apcin and proTAME could promote apoptosis in multiple myeloma cells [57]. While apcin, TAME, proTAME and apcin-A have shown an inhibitory role in suppressing Cdc20-APC/C function, their limited efficacy in killing cancer cells remains a challenge for clinical translation. Our development of CP5V based on PROTAC technology presents a new approach for targeting Cdc20. The observed potency of CP5V in inhibiting breast cancer cell growth and inducing cancer cell death at 2 μM level demonstrates its potential utility for anti-mitotic therapy. In the present work, we also observed that CP5V could resensitizes tamoxifen-resistant breast cancer cells to tamoxifen treatment. Although the underlying mechanism by which CP5V resensitizes endocrine resistance is currently unclear, we plan to further test if Cdc20 plays role in modulating estrogen receptor signaling or epigenetic modifiers involving in endocrine response [58], [59].

Progress in recent clinical trials on PROTAC drugs that target estrogen receptor, androgen receptor and BET family members provides strong hope for targeted protein degradation therapy [60], [61]. In such cases, the PROTACs have shown impressive efficiency in degrading target proteins and inducing cancer cell death at a nanomolar level. The oral estrogen receptor PROTAC, ARV-471, shows more robust tumor growth inhibition and ERα degradation compared to fulvestrant in an orthotopic MCF7/estradiol xenograft model, and inhibits growth of tamoxifen-resistant tumors while also reducing tumor ERa levels. While CP5V demonstrates a robust ability in degrading Cdc20, altering cyclin B levels, inducing mitotic arrest and promoting cancer cell death, the working concentration at 2 μM for CP5V still needs to be improved, and there are several strategies to improve the potency of anti-Cdc20 PROTAC. CP5V was designed based on apcin-A with a medium binding affinity. Lead optimization methods could be used to develop more potent Cdc20 inhibitors (Supplementary Figure 5). Apcin and apcin-A have similar binding affinities^14^ and they do not interact directly with the polar residues around the D-box binding sites (Supplementary Figure 5a). The low binding affinity of apcin-P indicates that the aromatic group of the pyrimidine ring in apcin (and apcin-A), which interacts with Trp209, is important to enable stronger affinity^14^. Based on the detailed analysis of the D-box binding pocket and its interactions with the known inhibitors, we can alter selected moieties of the known ligands to form enhanced interactions with Cdc20. Toward this goal, we have initiated an *in silico* high-throughput screening using pharmacophore model-based search against MolPort, ZINC and PubChem databases containing millions of compounds^38^. Three hits with enhanced interactions with the D-box binding pocket resulting from these simulations are illustrated in Supplementary Figure 5b–d, which could be explored for designing more potent Cdc20 inhibitors. In this study, although VHL/VBC E3 ligase showed an advantage over CRBN, other E3 ligases (such as MDM2 and IAP) could be considered in the design of PROTACs for targeting Cdc20. In addition, the design of the linker is another critical step that enables the suitable geometry and optimal conformational flexibility of the Cdc20-CP5V-VHL/VBC ternary complex. The linker length is important since the inter-residue interactions between VHL and Cdc20 can be better modulated with CP5V having an optimized length of linker (PEG5). Such considerations and efforts will be further pursued to design a high-potency PROTAC that targets Cdc20, building on the advances accomplished in the present study.

In summary, we report a novel strategy to efficiently target Cdc20-APC/C by designing a potent Cdc20 degrader via PROTAC platform using the VHL/VBC E3 ligase. This work advances the concept of targeting oncogenic proteins for degradation by designing PROTACs that specifically recruit endogenous ubiquitin protein ligases. Given the impact of Cdc20 in controlling mitosis, the development of CP5V provides a novel opportunity for anti-mitotic therapy.

## 5 Author contributions

Y.W. is the PI who supervized the whole project. Y.W., J.C., Z.Z., H.L., I.B., J.I. and Y.X. designed the experiments and writed original draft. J.C. built the conceptualization and performed the experiments. H.L. performed the molecular simulation of ternary complex formation and formal analysis. Z.Z. conducted the animal experiment and other experiments. J.I. synthesized the PROTAC molecules. G.E.S. supervised J.I.’s PROTACS synthesis. Y.X. conducted the bioinformatics analysis and data curation of clinical data under X.L.’s supervision. I.B. supervised H.L. in molecular modeling of the ternary complex. B. Z. and M.C. participated TCGA data analyses and IHC works. Y.W., I.B. and G.E.S. reviewed and edited the final draft.

## Funding sources

This work was supported by Northwestern University Zell scholar fund (YW), National Institutes of Health(NIH) grant # P41 GM103712 (IB), P30 CA060553 (ChemCore) and Chicago Biomedical Consortium with support from The Searle Funds at The Chicago Community Trust (ChemCore).

## Declaration of Competing Interest

Dr. Yong Wan, Dr. Zhuan Zhou, Mr. Junlong Chi and Dr. Gary Schiltz are inventors on pending patent Application #62/834,550.

## Appendix Supplementary materials

Image, application 1

Image, application 2

Image, video 3
