# Supplementary material for: A novel strategy to block mitotic progression for targeted therapy
Source: eBioMedicine. 2019 Oct 25;49:40–54. doi: 10.1016/j.ebiom.2019.10.013 (PMC6945239; doi:10.1016/j.ebiom.2019.10.013)
Supplement: Supplementary file 1 [file mmc1.docx]

**Supplementary Figure 1**


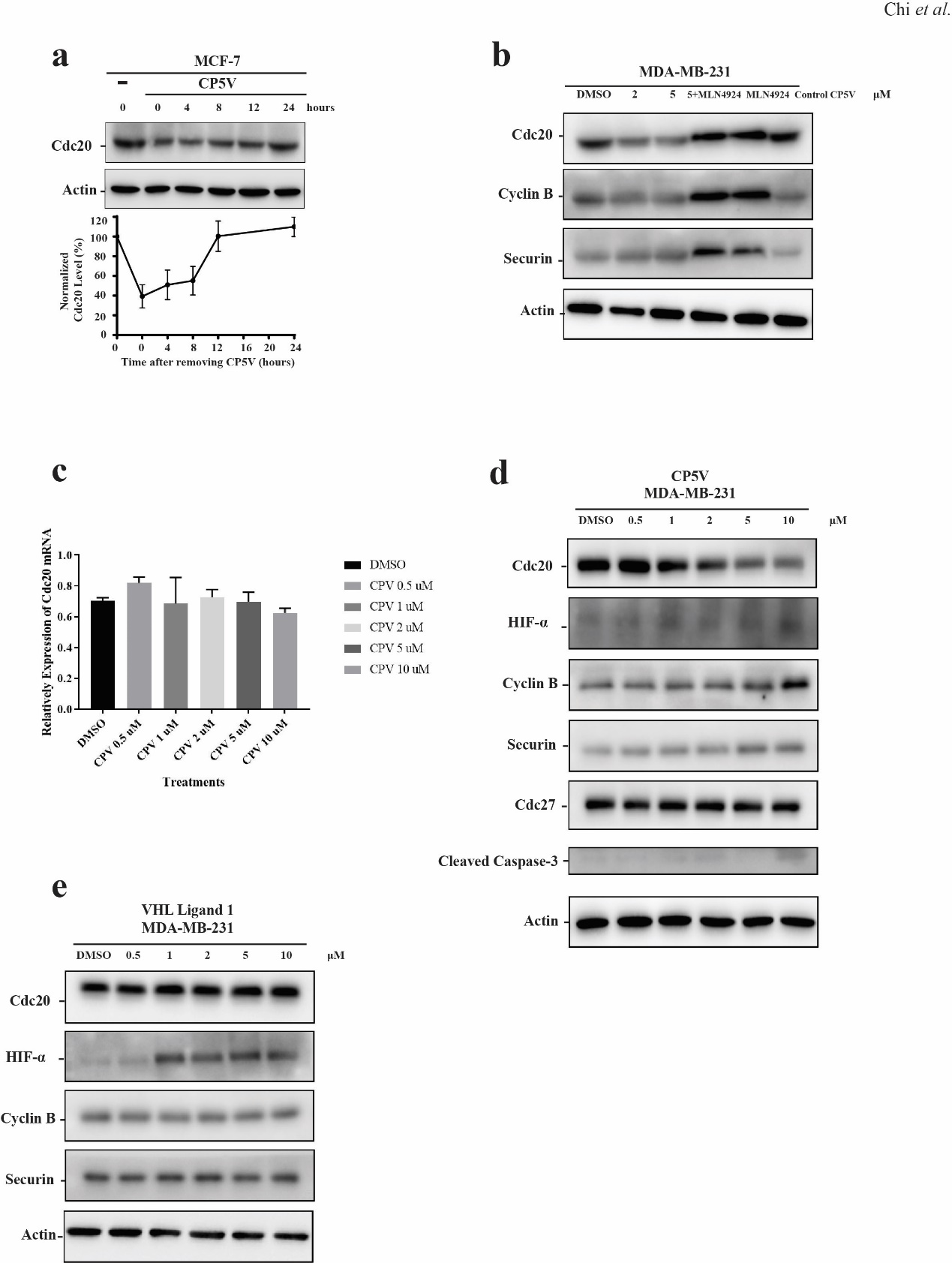


**Supplementary Figure 1. CP5V causes ubiquitin-proteasome dependent selective degradation of Cdc20.** (**a**) The MCF-7 cells were treated with CP5V at 2 μM for 8 hours followed by a release in fresh medium. Cells were collected at different time points (0, 4, 8, 12, 24 hours) for measuring Cdc20 protein levels by Western blotting. The assays for a-h were measured in triplicate (*n* = 3). Data are mean ± SEM. (**b**) MLN4924 inhibits CP5V-mediated Cdc20 degradation. MDA-MB-231 cells were treated with CP5V or CP5V and MLN4924 (5 μM) at the indicated dose for 10 hours. Cdc20, cyclin B, securin, and actin levels were determined by Western blotting. (**c**) Relative RNA expression in MDA-MB-231 cells treated with CP5V. MDA-MB-231 cells treated with CP5V at indicated dosages for 10 hours were harvested for RNA extraction, reverse-transcription, and further qRT-PCR. (**d**) CP5V caused selective degradation of Cdc20 without causing an apparent accumulation of HIF-α in MDA-MB-231 cells. MDA-MB-231 cells treated with CP5V at indicated dosages for 10 hours were harvested for Western Blot. (**e**) VHL Ligand 1 induced HIF-α accumulation with a concentration higher equal or than 1 μM in MDA-MB-231 cells. MDA-MB-231 cells treated with VHL Ligand 1 at indicated dosages for 10 hours and collected for WB.

**Supplementary Figure 2**


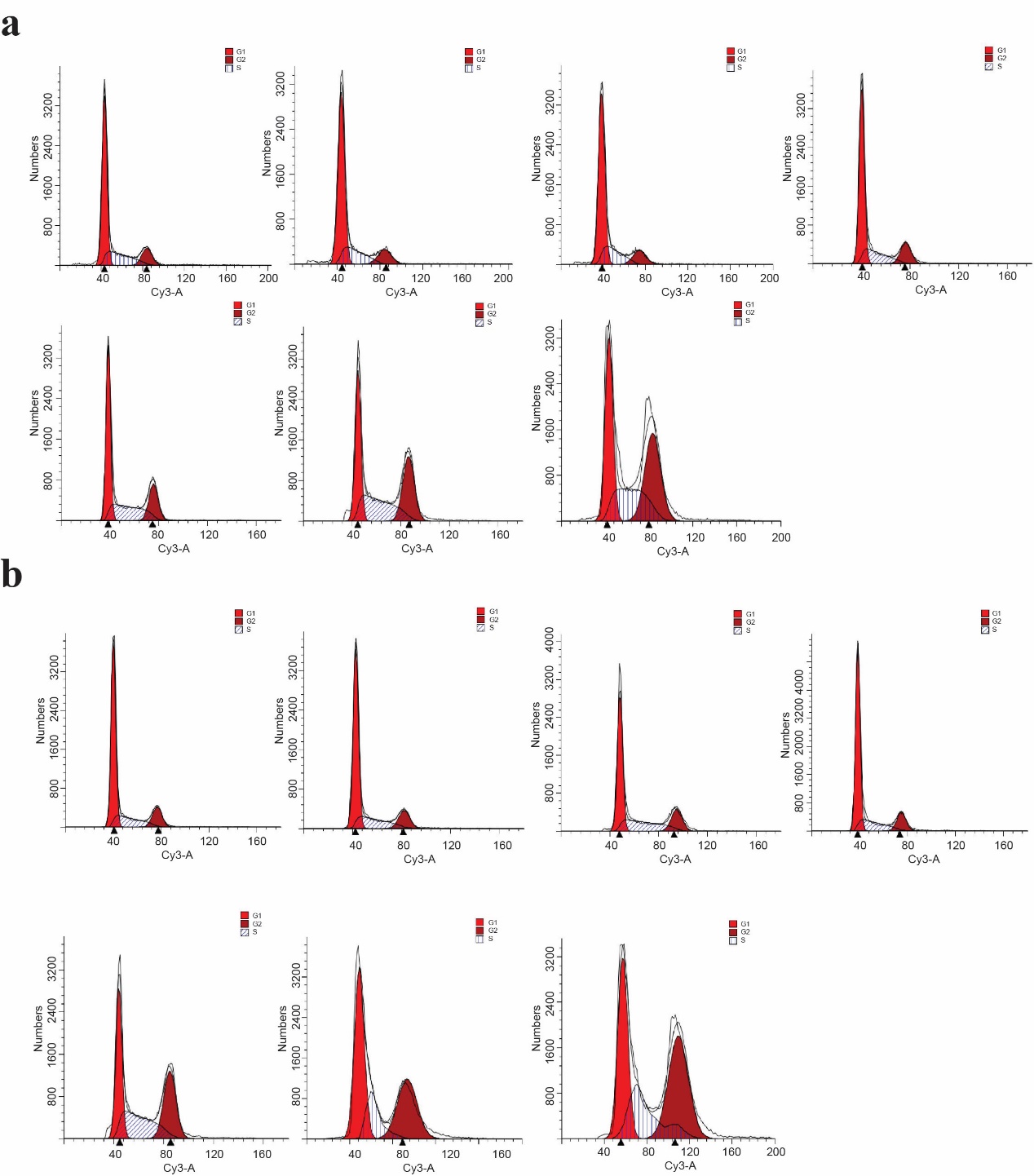


**Supplementary Figure 2. The original flow cytometry data for figure 4C.** (**a**) MDA-MB-231 cells and (**b**) MDA-MB-435 cells were initially synchronized by double-thymidine treatment followed by treatment with CP5V for 16 hours. Cell cycle profile was then measured by flow cytometry and analyzed by ModFit LT. The order of the results is: No treatment, DMSO, apcin (10 μM), control CP5V (10 μM), CP5V (1, 2, and 5 μM).

**Supplementary Figure 3**

**
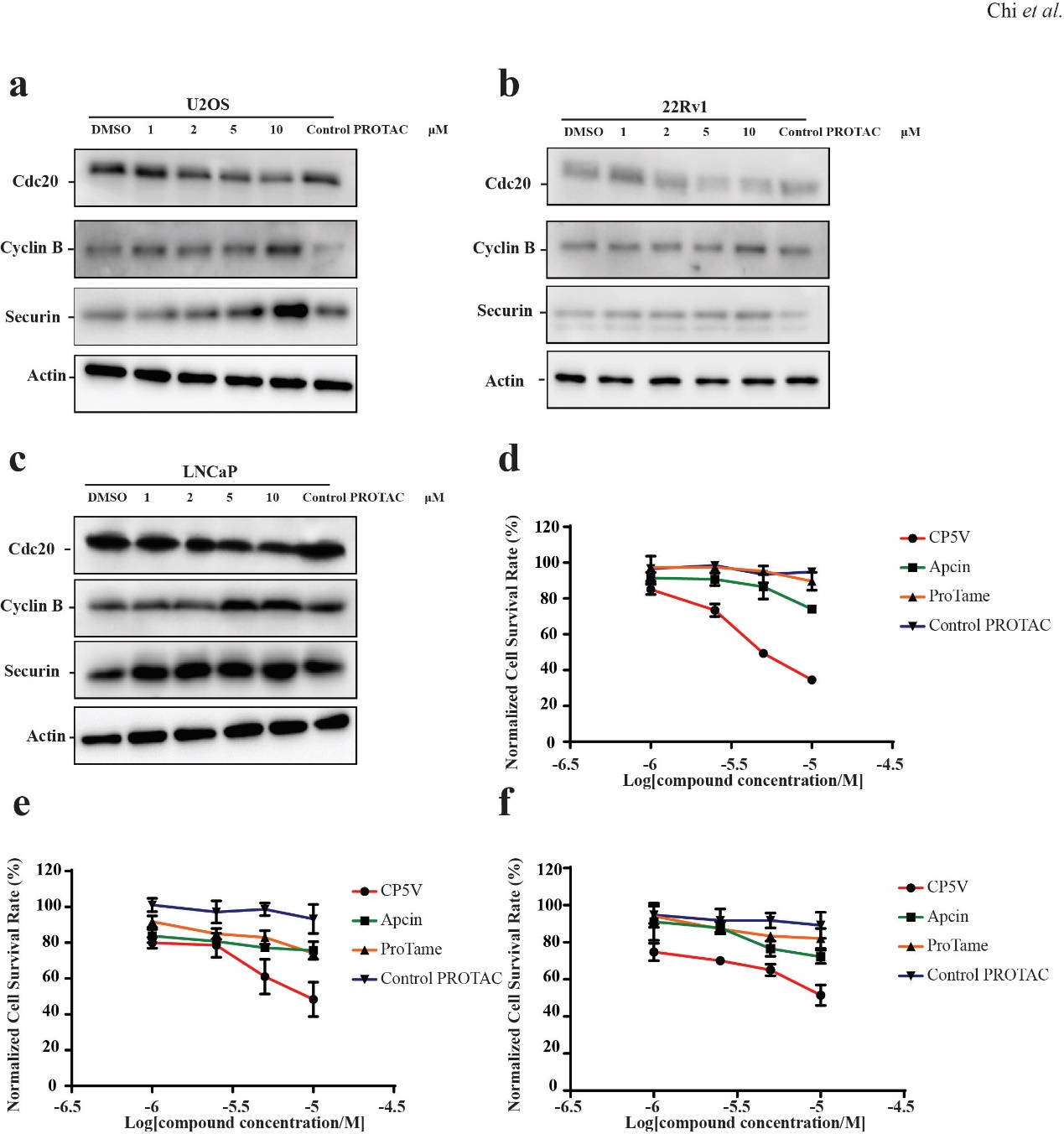
**

**Supplementary Figure 3. CP5V can induce Cdc20 degradation and growth inhibition in other cancer cell lines.** Dosage dependency of CP5V action in (**a**) U2OS (Osteosarcoma cell line), (**b**) 22Rv1, and (**c**) LNCaP cells (Prostate cancer cell lines). The cancer cells were treated with CP5V with indicated dosages for 10 hours and collected for WB. CP5V causes significant inhibition of cell growth in (**d**) U2OS, (**e**) 22Rv1, and (**f**) LNCaP cells. The cancer cells were plated in 96-well plates at the concentration of 3000 cells/well and treated with DMSO, apcin, ProTame, and CP5V for 72 hours and the cell survival activity was measured by CCK8 assay. The test was performed in triplicate (*n* = 3). Data are mean ± SEM.


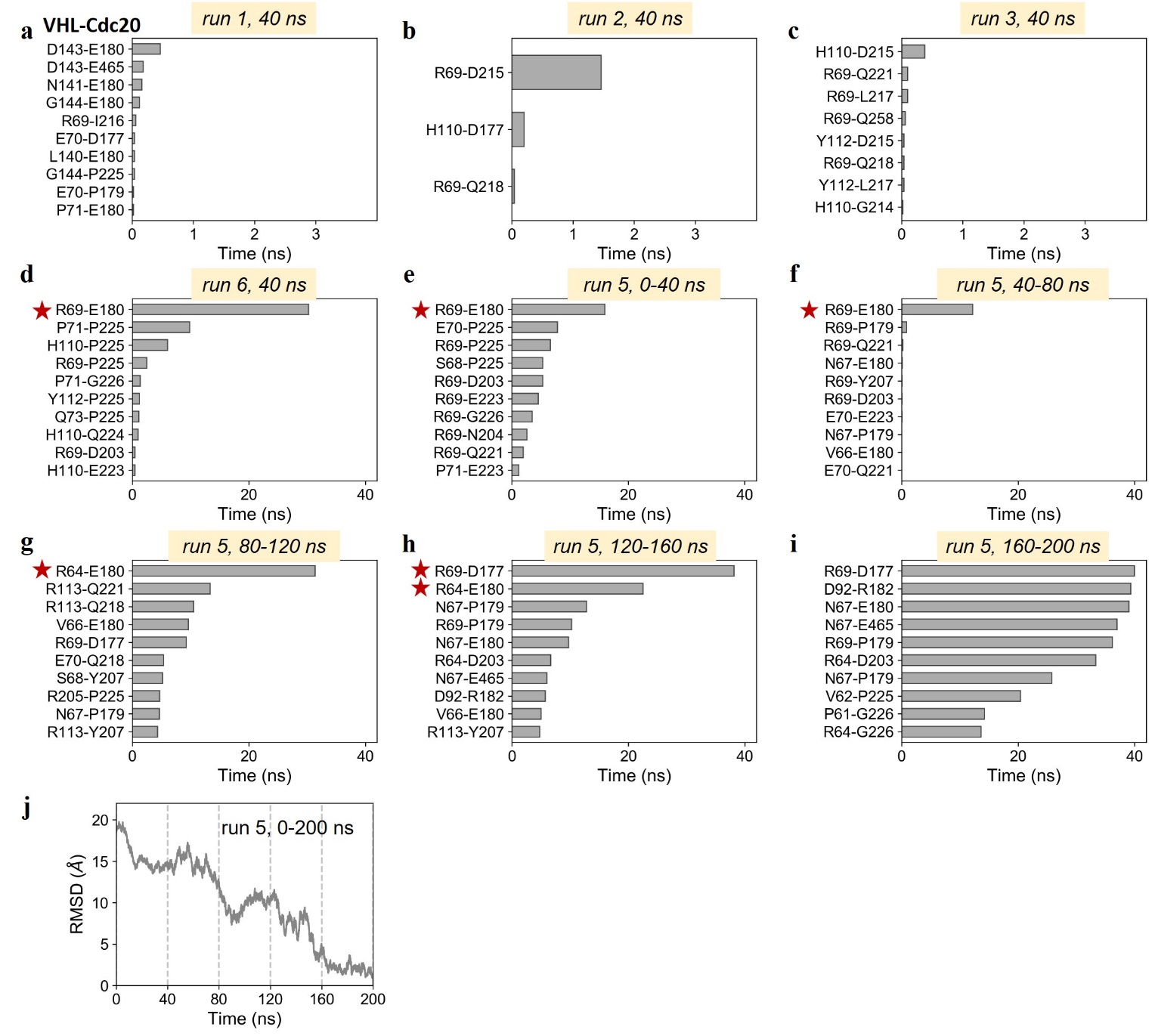
**Supplementary Figure 4**

**Supplementary Figure 4. Intermolecular residue-residue interactions between VHL and Cdc20 observed in six independent MD simulations.** Simulations were performed for the pair of proteins VHL-Cdc20 linked by CP5V, starting from different initial orientations**.** The plots display the total time during which the residue pairs indicated along the ordinate made intermolecular contacts. Contacts are defined when residue pairs (*left* for VHL and *right* for Cdc20) have any two heavy atoms separated by less than 4.0 Å. Results are displayed for runs (**a**) 1, (**b**) 2, (**c**) 3 and (**d**) 6, each of 40 ns. We also performed a long run of 200 ns (*run 5*) displayed in five panels: (**e**) 0-40 ns, (**f**) 40-80 ns, (**g**) 80-120 ns, (**h**) 120-160 ns and (**i**) 160-200 ns. Up to 10 most frequent interactions are displayed in panels **a**-**i**. Seven top-ranking residue-residue interactions shown in panel **i** played a key role in stabilizing the final conformer. Results from *run 4* are not shown since the apcin-A fragment of CP5V got detached from the D-box binding site of Cdc20*. Red stars* indicate residue-residue interactions that were transiently stabilized during simulations.

**Supplementary Figure 5**


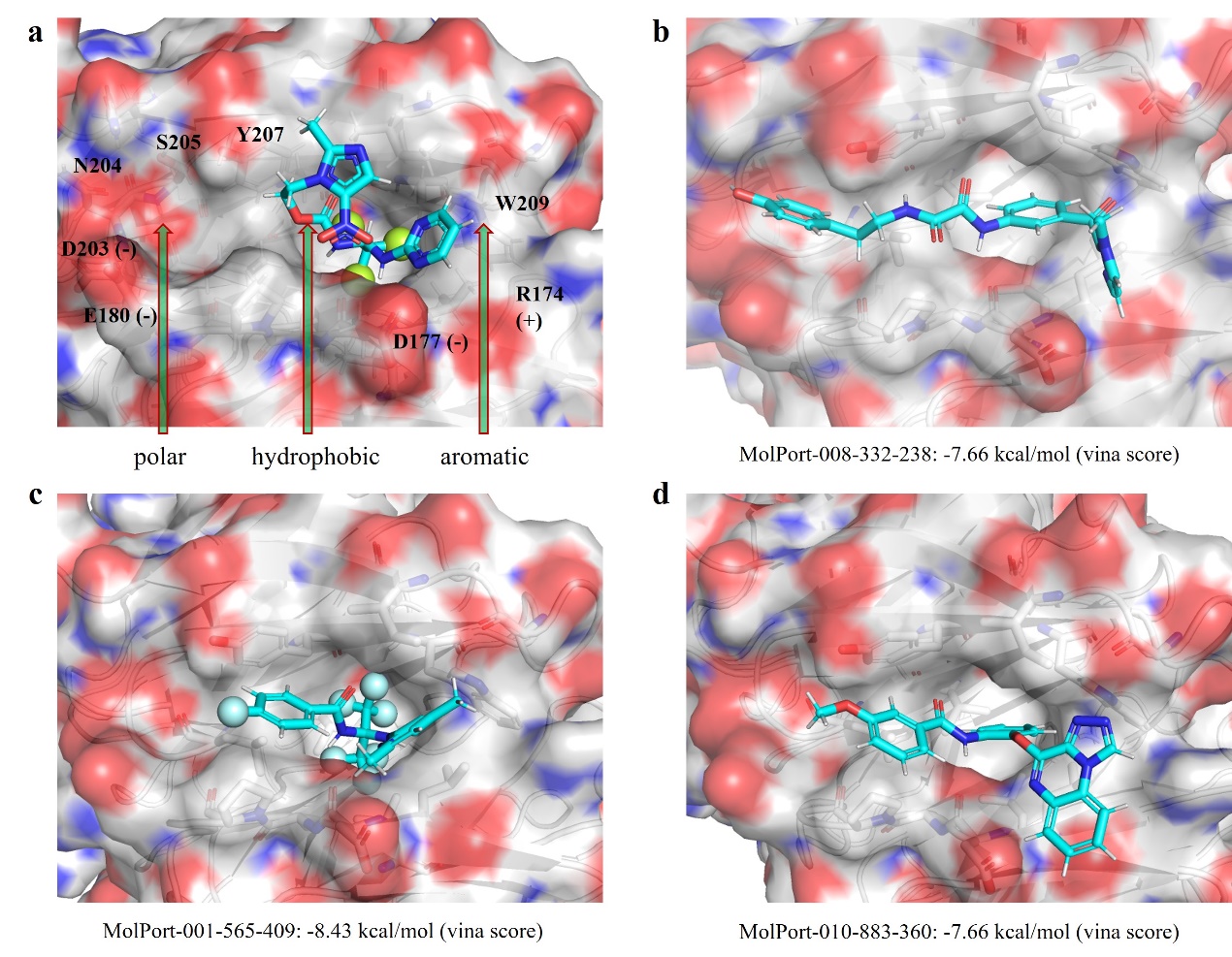


**Supplementary Figure 2. Toward designing more potent PROTACs: high-throughput screening and lead optimization of Cdc20 inhibitors.** (**a**) Apcin binds to the D-box binding pocket of Cdc20 (PDB id: 4n14). A deep small hydrophobic pocket is occupied by the trichloromethyl group and the pyrimidine has a week π-π interaction with W209. Three features (polar, hydrophobic and aromatic) indicated with arrows could be enhanced for designing ligands with a higher binding affinity. C-, N- and O-atoms of the protein are colored *white*, *blue* and *red*, respectively. Docking poses of three purchasable compounds. (**b**) N'-[2-(4-hydroxyphenyl)ethyl]-N-[3-(1-methyl-1H-imidazole-2-carbonyl)phenyl]ethanediamide, (c) N-{2-[(4,6-dimethylpyridin-2-yl)amino]-1,1,1,3,3,3-hexafluoropropan-2-yl}-4-fluorobenzamide and (d) 3-methoxy-N-(2-{[1,2,4]triazolo[4,3-a]quinoxalin-4-yloxy}phenyl)benzamide are shown. The corresponding binding affinities (Vina scores) are indicated. The compounds were obtained by screening our pharmacophore model against the MolPort database of small compounds ([https://www.molport.com](https://www.molport.com/)) using the Pharmit^1^. These three compounds enhance the polar (H-bonds), hydrophobic and π-π (cation-π) interactions indicated in (a), respectively. The Cl and F atoms in the compounds are highlighted in *yellow-green* and *light-cyan* spheres.

**REFERENCE**

1. Sunseri J, Koes DR. Pharmit: interactive exploration of chemical space*,*Nucleic Acids Res*,* 2016 July 8; **44**(W1): W442-448
