# Supplementary material for: A novel strategy to block mitotic progression for targeted therapy
Source: eBioMedicine. 2019 Oct 25;49:40–54. doi: 10.1016/j.ebiom.2019.10.013 (PMC6945239; doi:10.1016/j.ebiom.2019.10.013)
Supplement: Supplementary file 2 [file mmc2.pdf]

Supplementary method for synthesis of CP5V (VHL Ligand 1-PEG5-apcin-A)

VHL Ligand 1-PEG5-apcin-A

((S)-25-((2S,4R)-4-hydroxy-2-((4-(4-methylthiazol-5-yl)benzyl)carbamoyl)pyrrolidine-1-carbonyl)-26,26-dimethyl-5,23-dioxo-8,11,14,17,20-pentaoxa-4,24-diazaheptacosyl (2,2,2-trichloro-1-(pyrimidin-2-ylamino)ethyl)carbamate)

Step 1

To a 2 ml vial containing 4,7,10,13,16-pentaoxanonadecanedioic acid (629 mg, 2 Eq, 1.86 mmol) was added DMF (3 mL) and the mixture was cooled in an ice/water bath. Then, HATU (353 mg, 1 Eq, 929 µmol) and DIPEA (480 mg, 0.65 mL, 4 Eq, 3.72 mmol) were added. The mixture was stirred in the cold bath for 15 min after which (2S,4R)-1-((R)-2-amino-3,3-dimethylbutanoyl)-4-hydroxy-N-(4-(4-methylthiazol-5-yl)benzyl)pyrrolidine-2-carboxamide (400 mg, 1 Eq, 929 µmol) in DMF (0.2 mL) was added. The reaction mixture was allowed to stir overnight as the ice bath expired for 16 h after which LC/MS indicated formation of product. The mixture was directly purified by RP HPLC eluting with 5 to 50 % acetonitrile in water (0.1 % Formic acid modifier) to give (S)-21-((2S,4R)-4-hydroxy-2-((4-(4-methylthiazol-5-yl)benzyl)carbamoyl)pyrrolidine-1-carbonyl)-22,22-dimethyl-19-oxo-4,7,10,13,16-pentaoxa-20-azatricosanoic acid (440 mg, 63.1 %) as a light yellow oil. LC/MS, NMR CMIDD-159-1-2.

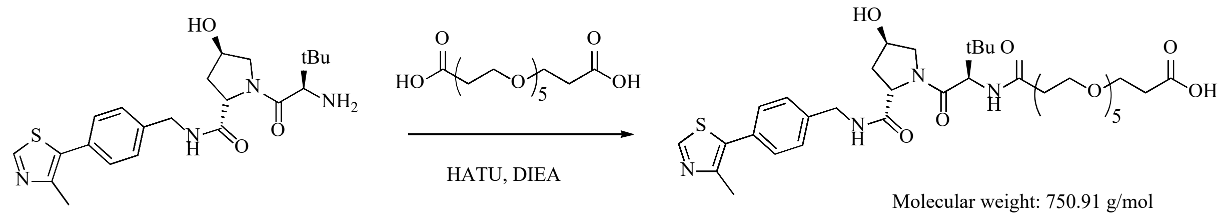

Molecular weight: 750.91 g/mol

LC/MS

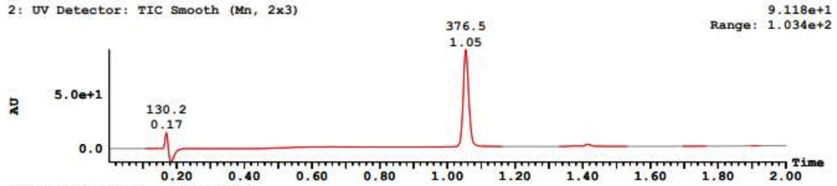

Time	Mass Found	Area %Total
0.13		0.01
0.17		3.17
0.25		27.17
1.05		68.86
1.38		0.22
1.41		0.53
1.50		0.01
1.72		0.01
1.75		0.01
1.91		0.01

Time	Mass Found
1.07	

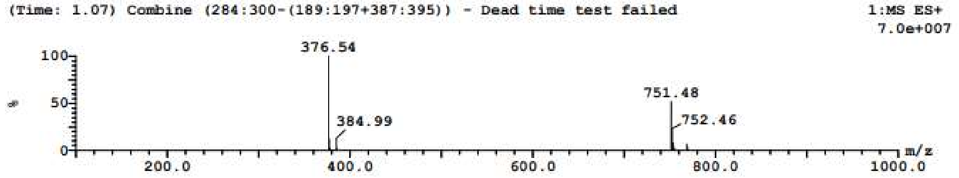

1H NMR

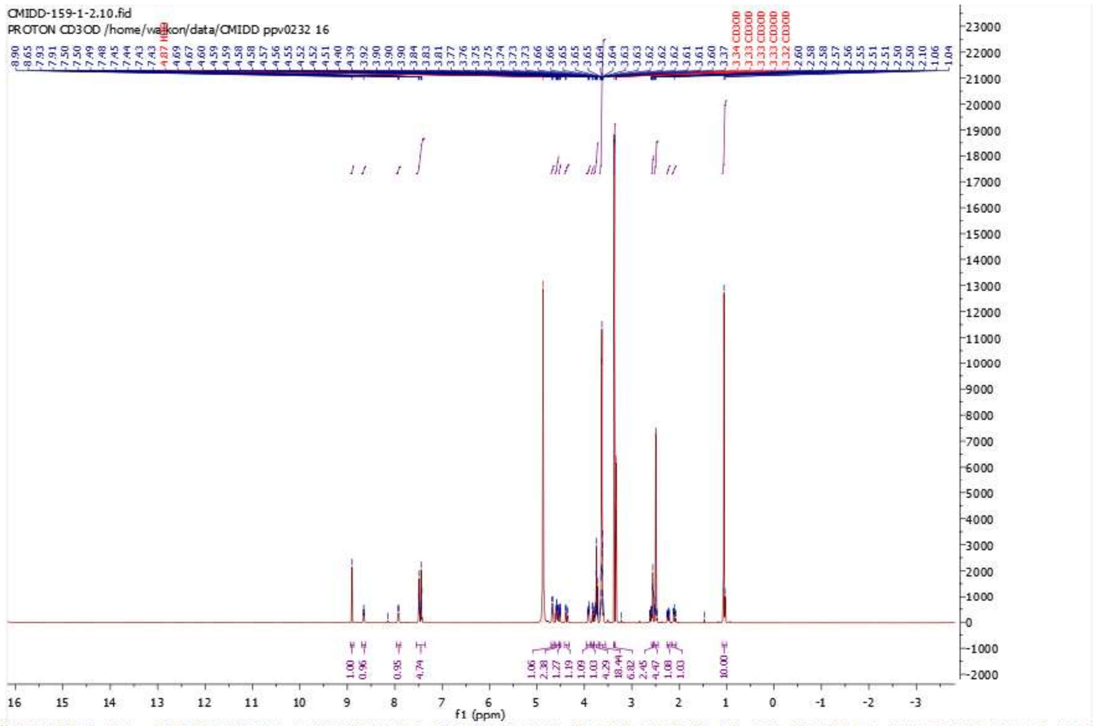

^{1}H NMR (500 MHz, Methanol-d_4) δ 8.90 (s, 1H), 8.65 (t, J = 6.1 Hz, 1H), 7.92 (d, J = 9.0 Hz, 1H), 7.54 – 7.36 (m, 5H), 4.68 (d, J = 9.0 Hz, 1H), 4.62 – 4.54 (m, 2H), 4.52 (qd, J = 4.9, 4.1, 2.7 Hz, 1H), 4.38 (dd, J = 15.4, 5.4 Hz, 1H), 3.91 (dt, J = 11.2, 1.7 Hz, 1H), 3.82 (dd, J = 11.0, 3.9 Hz, 1H), 3.80 – 3.70 (m, 4H), 3.68 – 3.55 (m, 18H), 3.37 (s, 7H), 2.56 (t, J = 6.2 Hz, 2H), 2.50 (s, 4H), 2.24 (ddt, J = 11.6, 7.6, 2.0 Hz, 1H), 2.10 (ddd, J = 13.3, 9.0, 4.5 Hz, 1H), 1.06 (s, 10H).

13C NMR

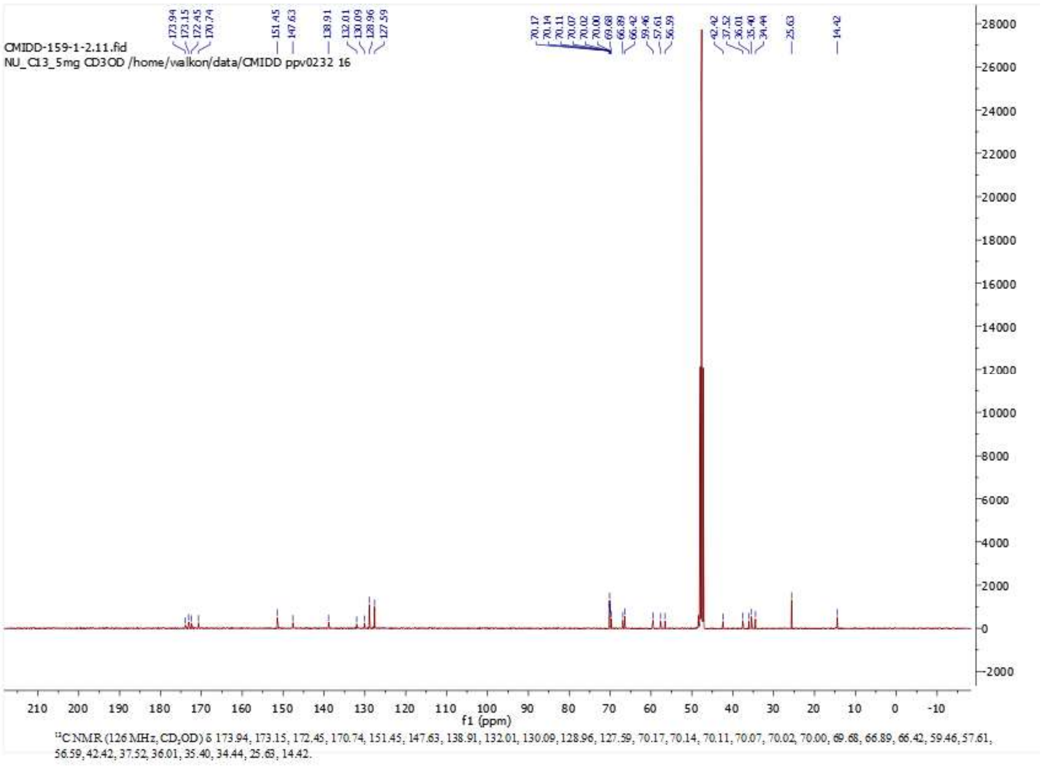

^{13}C NMR (126 MHz, CD_3OD) δ 173.94, 173.15, 172.45, 170.74, 151.45, 147.63, 138.91, 132.01, 130.09, 128.96, 127.59, 70.17, 70.14, 70.11, 70.07, 70.02, 70.00, 69.68, 66.89, 66.42, 59.46, 57.61, 56.59, 42.42, 37.52, 36.01, 35.40, 34.44, 25.63, 14.42.

Step 2

To a solution of (R)-21-((2S,4R)-4-hydroxy-2-((4-(4-methylthiazol-5-yl)benzyl)carbamoyl)pyrrolidine-1-carbonyl)-22,22-dimethyl-19-oxo-4,7,10,13,16-pentaoxa-20-azatricosanoic acid (200 mg, 1 Eq, 266 µmol) in DMF (2 mL) in an ice/water bath was simultaneously added 3-aminopropyl (2,2,2-trichloro-1-(pyrimidin-2-ylamino)ethyl)carbamate [apcin-A] (183 mg, 2 Eq, 533 µmol) and HATU (253 mg, 2.5 Eq, 666 µmol). Right after addition, DIEA (138 mg, 186 µL, 4 Eq, 1.07 mmol) was added and the reaction mixture was stirred as the ice bath expired for 2 h after which LC/MS indicated product. The mixture was directly purified via RP HPLC eluting with 10 to 90 % acetonitrile in water (0.1 % formic acid modifier) to give (S)-25-((2S,4R)-4-hydroxy-2-((4-(4-methylthiazol-5-yl)benzyl)carbamoyl)pyrrolidine-1-carbonyl)-26,26-dimethyl-5,23-dioxo-8,11,14,17,20-pentaoxa-4,24-diazaheptacosyl (2,2,2-trichloro-1-(pyrimidin-2-ylamino)ethyl)carbamate (214 mg, 74.7 %) as a yellow oil. LC/MS, NMR CMIDD-159-6-2. Note: on
LC/MS, M/2 seen.

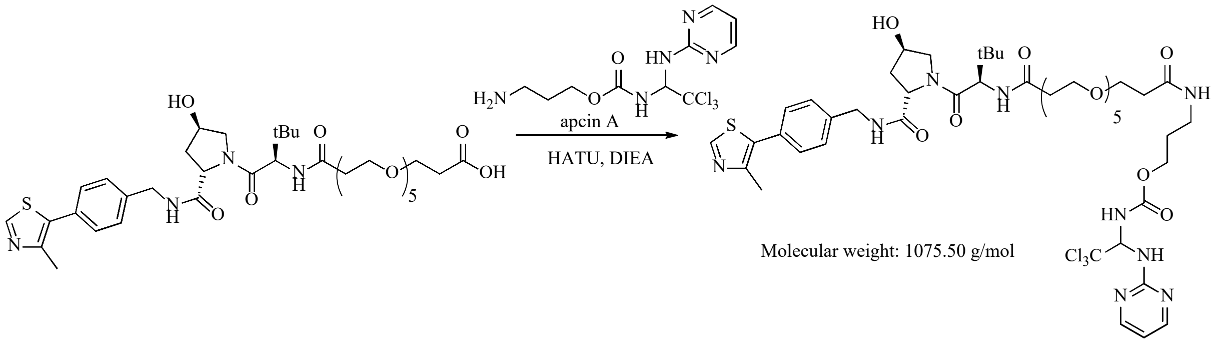

LC/MS

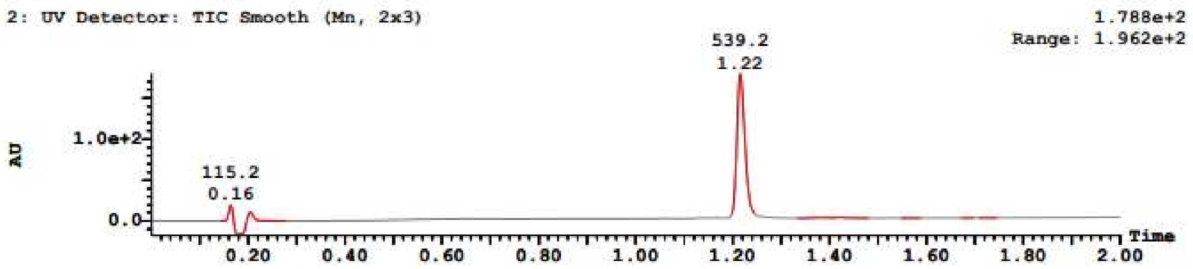

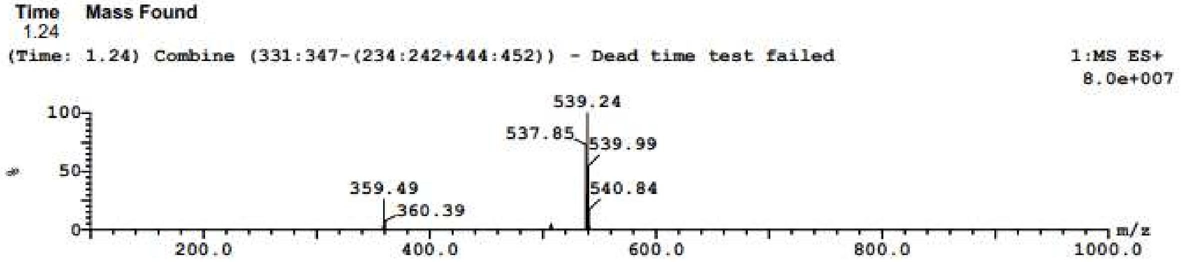

1H NMR

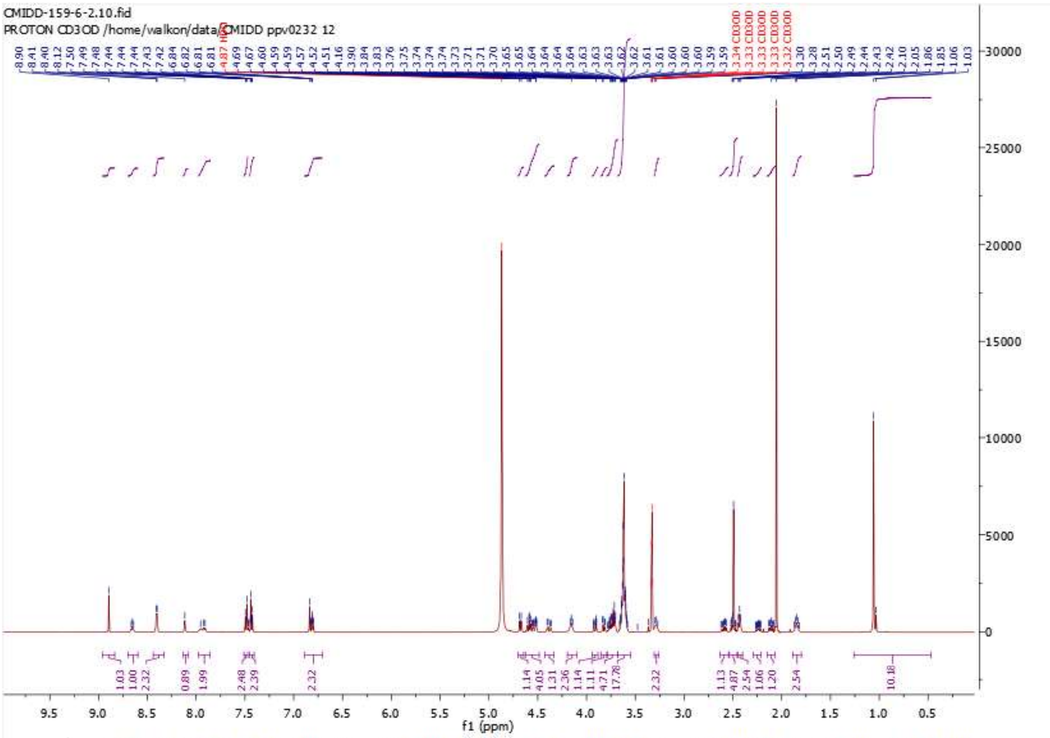

^{1}H NMR (500 MHz, Methanol-d_4) δ 8.90 (s, 1H), 8.65 (t, J = 6.0 Hz, 1H), 8.41 (d, J = 4.9 Hz, 2H), 8.12 (s, 1H), 7.98 – 7.86 (m, 2H), 7.49 (d, J = 8.2 Hz, 2H), 7.45 – 7.41 (m, 2H), 6.89 – 6.71 (m, 2H), 4.68 (d, J = 9.0 Hz, 1H), 4.63 – 4.48 (m, 4H), 4.38 (dd, J = 15.5, 5.2 Hz, 1H), 4.16 (t, J = 6.4 Hz, 2H), 3.91 (dt, J = 11.3, 1.8 Hz, 1H), 3.82 (dd, J = 11.0, 3.9 Hz, 1H), 3.79 – 3.68 (m, 5H), 3.68 – 3.55 (m, 18H), 3.28 (t, J = 6.7 Hz, 2H), 2.59 (ddd, J = 15.0, 7.5, 5.3 Hz, 1H), 2.54 – 2.46 (m, 5H), 2.43 (t, J = 6.0 Hz, 3H), 2.29 – 2.21 (m, 1H), 2.10 (ddd, J = 13.3, 9.1, 4.4 Hz, 1H), 2.07 (s, 0H), 1.85 (p, J = 6.4 Hz, 3H), 1.06 (s, 10H).

13C NMR

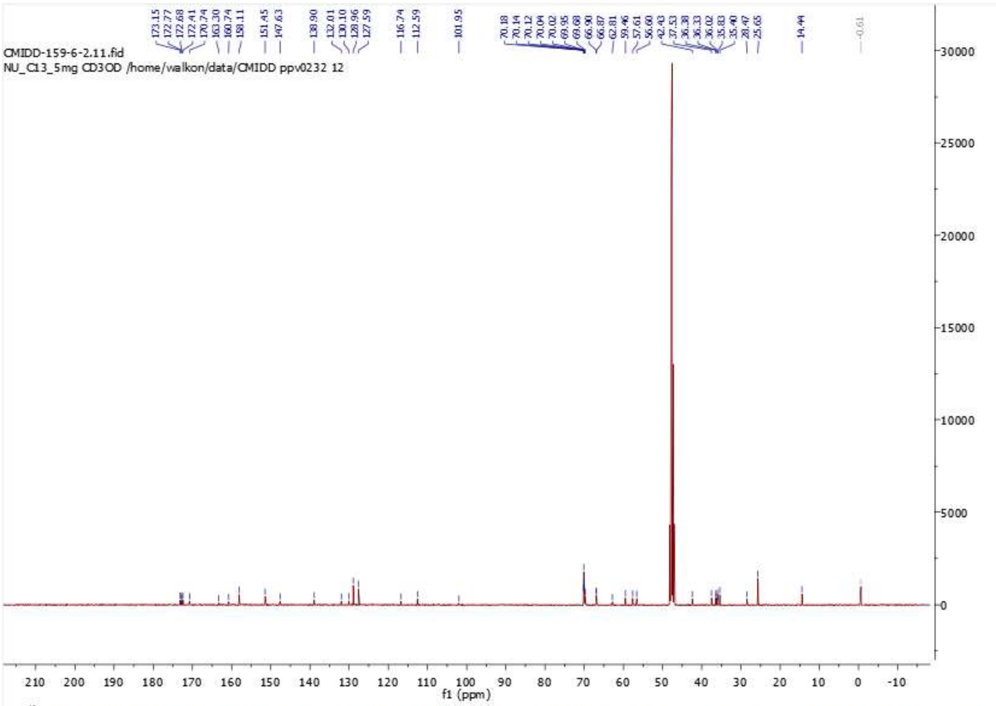

^{13}C NMR (126 MHz, CD_3OD) δ 173.15, 172.77, 172.68, 172.41, 170.74, 163.30, 160.74, 158.11, 151.45, 147.63, 138.90, 132.01, 130.10, 128.96, 127.59, 116.74, 112.59, 101.95, 70.18, 70.14, 70.12, 70.04, 70.02, 69.95, 69.68, 66.90, 66.87, 62.81, 59.46, 57.61, 56.60, 42.43, 37.53, 36.38, 36.33, 36.02, 35.83, 35.40, 28.47, 25.65, 14.44
